# Electron Trapping Prolongs the Lifetime of Charge-Separated States in 2D Perovskite Nanoplatelet-Hole Acceptor Complexes

**DOI:** 10.1021/acs.jpclett.2c03815

**Published:** 2023-02-23

**Authors:** Sheng He, Tao Jin, Anji Ni, Tianquan Lian

**Affiliations:** Department of Chemistry, Emory University, 1515 Dickey Drive Northeast, Atlanta, Georgia 30322, USA

## Abstract

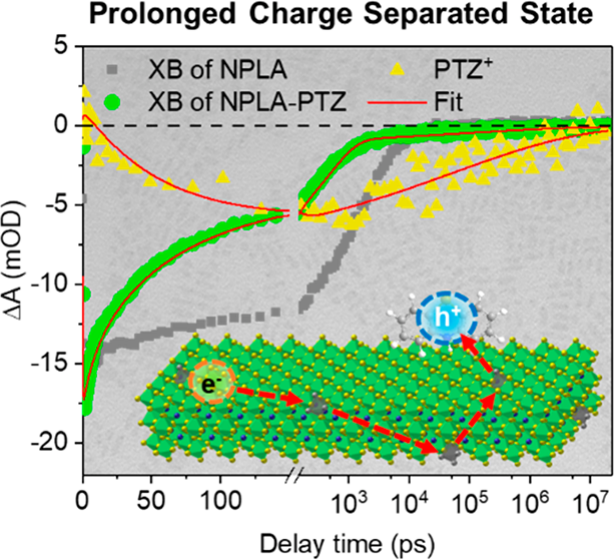

Two-dimensional (2D) lead halide perovskite nanoplatelets (NPLs) are promising materials for blue light emission because of the strong quantum confinement in the 2D morphology. However, the identity of carrier traps and the trap influence on charge transfer in these NPLs remain unclear. Herein, transient absorption studies revealed two types of electron traps in 3 monolayer lead bromide perovskite NPLs with trapping lifetime of 9.0 ± 0.6 and 516 ± 59 ps, respectively, while no hole traps were observed. Systematic charge transfer experiments show that electron traps have negligible influence on ultrafast electron transfer or hole transfer but extend the half-lifetime of the charge-separated state from 2.1 ± 0.1 to 68 ± 3 ns after hole transfer, which is explained by the reduced electron–hole overlap. This work contributes to the understanding of the fundamental carrier dynamics in 2D perovskite NPLs and offers guidelines for boosting their performance in optoelectronics and photocatalysis.

Lead halide perovskite APbX_3_ (A = Cs^+^, methylammonium, formamidinium; X = Cl^–^, Br^–^, I^–^) nanomaterials have captured intense research interest since their first successful colloidal synthesis.^1,2^ In addition to the properties of bulk perovskites, such as defect tolerance and ionic crystal lattice,^3−6^ the nanosized perovskites also benefit from size- and morphology-dependent quantum confinement effects,^7,8^ facile anion exchange,^9−11^ and multifunctional surface modifications,^12−17^ showing great promise in applications in light-emitting diodes (LEDs),^18−20^ solar cells,^21,22^ and photocatalysis.^23−28^ Among various perovskite nanomaterials, two-dimensional (2D) lead bromide perovskite nanoplatelets (NPLs) with a stoichiometric formula of L_2_Cs_*n*–1_Pb_*n*_Br_3*n*+1_ (L: ammonium ligand) are gaining intense attention because of their efficient photoluminescence (PL) in the blue region caused by the strong quantum confinement along the NPL thickness direction.^29−37^ The low dimensionality of 2D perovskite NPLs, on the other hand, inevitably introduces surface defects, degrading their performance in light-emitting applications.^29,33,35^ Although several surface treatments have been reported to passivate the NPL surface defects with the aim of improving the PL quantum yield (PLQY),^33,35,37,38^ the carrier trap identity (electron or hole trap) or the passivation mechanism is still unclear.

Although often regarded as the main source of nonradiative recombination and low PLQY in semiconductor nanocrystals (NCs), charge carrier trapping has been shown to promote charge separation in prototypical cadmium chalcogenide NCs. For example, hole trapping, induced by undercoordinated chalcogen atoms,^39,40^ has been shown to decouple the electron and hole wave functions to promote electron transfer (ET) to acceptors and to extend the charge-separated (CS) state lifetime in CdS and CdSe nanorods (NRs) or NPLs.^41−44^ The localized hole in the trap state can also mediate indirect hole extraction in CdSe-based quantum dots (QDs).^45,46^ Multiple hole extraction from CdSe QDs is also realized by hole trap states which are decoupled from the valence band (VB) and prevent Auger recombination.^47^ In addition, hole traps can also mediate triplet energy transfer in CdSe, PbS, and CuInS_2_ QDs.^48−51^ However, similar effects of trap states on charge transfer in perovskite NCs have not been reported. One possible reason is the defect tolerance, especially in bromide- and iodide-based cuboidal perovskite NCs, where the defect-induced trap states locate within the conduction band (CB) and VB or near the band edges,^14,52−54^ resulting in negligible changes to the electron–hole wave function overlap in the excited state. On the other hand, 2D perovskite NPLs and chloride-based cuboidal perovskite QDs may suffer from in-gap deep traps, as suggested by their low PLQY and the necessity of postsynthesis surface passivation for their blue light-emitting applications.^33,35,55−57^ Although charge transfer from perovskite NCs has been studied because of its importance in photovoltaics and photocatalysis applications,^58−61^ how the trap states in these NCs affect the charge extraction performance remains unexplored.

In this work, we use surface passivation to control trap states in three monolayer (ML) L_2_Cs_*n*–1_Pb_*n*_Br_3*n*+1_ (*n* = 3) perovskite NPLs and to investigate the surface passivation mechanism and the impact of carrier traps on interfacial charge transfer. The trap identity is investigated by ultrafast spectroscopies, and the impact of carrier traps on charge transfer is systematically studied using selected charge acceptors. Transient absorption (TA) studies of the 3 ML NPLs with and without surface passivation unveil two types of electron traps and negligible hole traps. The electron traps show no influence on ultrafast ET and hole transfer (HT) to selected charge acceptors, or interfacial exciton dissociation, but extend the CS state lifetime by at least 1 order of magnitude in the HT case. The electron trap-induced long-lived CS state is understood by the long electron–hole distance and decreased electron–hole wave function overlap imposed by the 2D morphology of the NPLs. The knowledge obtained here offers not only rational guidelines for improving 2D perovskite NPLs in optoelectronic and photocatalysis applications but also insights into the fundamental carrier dynamics in low-dimensional perovskite materials.

Three ML L_2_Cs_*n*–1_Pb_*n*_Br_3*n*+1_ NPLs are synthesized following a literature procedure with slight modification,^33,62^ the details of which can be found in the Supporting Information, section S1. Transmission electron microscopy (TEM) images (Figure S1) of the 3 ML NPLs show a rectangular shape (15.7 ± 2.7 nm long, 5.8 ± 0.9 nm wide; Figure S2), consistent with previous reports.^33,63^ To passivate the as-synthesized NPLs (referred to as NPLAs hereafter), a toluene solution of PbBr_2_, oleic acid, and oleylamine is added into the colloidal NPLs, as shown in Figure 1a.^62^ Both Pb^2+^ and Br^–^ are found necessary to enhance the PL intensity of L_2_Cs_*n*–1_Pb_*n*_Br_3*n*+1_ NPLs, and the organic ligands help to dissolve the PbBr_2_ salt and stabilize the colloidal NPLs.^33,35^ The size and morphology of the NPLs are retained after the surface passivation process (Figures S1 and S2). The passivated NPLs (referred to as NPLEs hereafter) show enhanced PL, exhibiting bright blue emission even under ambient room light (Figure 1b).

**Figure 1:**
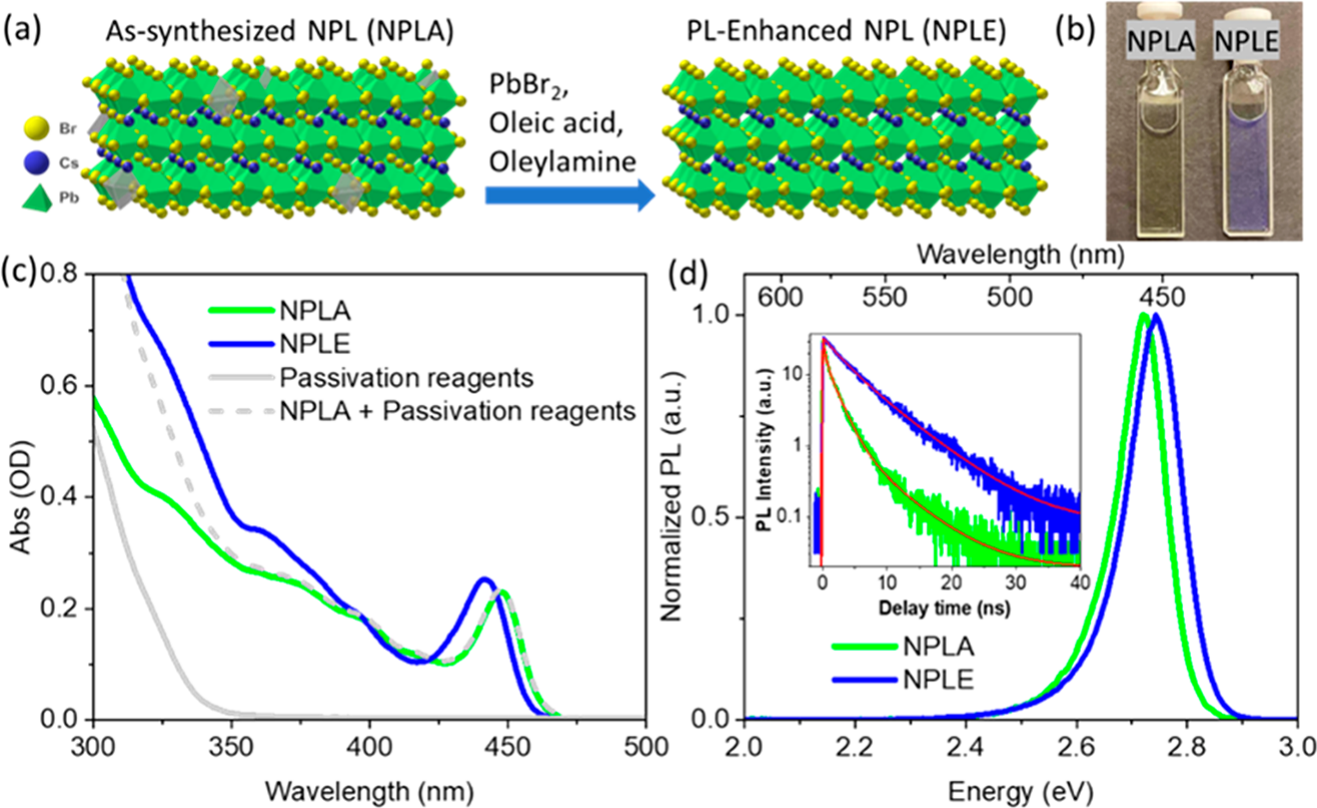
Passivation of NPLs. (a) Schematic of the passivation method that converts NPLAs to NPLEs. The gray octahedron in the lattice of NPLA represents the Pb^2+^ vacancy. (b) Optical image of the NPLA and NPLE samples under ambient room light. (c) Absorption spectra of the NPLA, NPLE, and the passivation reagents. The gray dashed line shows the sum of the absorption of NPLA and passivation reagents. (d) PL spectra of the NPLA and NPLE samples. The inset shows PL decay curves normalized by number of absorbed photons. The red lines represent multiexponential fits to the data.

The UV–vis absorption spectrum of the NPLA (Figure 1c) shows a band edge exciton transition peak at 448 nm, agreeing well with reported values in 3 ML lead bromide perovskite NPLs.^33,64,65^ The NPLE sample shows a slightly blue-shifted exciton peak at 441 nm, suggesting that the passivation process does not change the NPL thickness (or the number of layers) but increases the dielectric confinement effect (see the Supporting Information, section S3 for more details).^8,66,67^ At wavelengths shorter than 400 nm, the NPLE sample also shows an increase in absorbance due to the presence of excess passivation reagents (lead oleate, oleylammonium bromide, gray curve in Figure 1c). In addition, the difference between the NPLE spectrum and the sum of NPLA and passivation reagents spectra (gray dashed curve in Figure 1c) in this region is attributed to NPL volume growth in the lateral direction after passivation, consistent with the TEM results (Figure S1a,d). Comparison of the normalized PL spectra of NPLA and NPLE in Figure 1d shows that the PL peak shifts from 456 to 452 nm after passivation, consistent with the absorption peak shift. The PL spectra show asymmetric peak shapes, which have been attributed to the formation of self-trapped excitons that have a lower transition energy than the band edge exciton.^68^ The PL lifetime (inset in Figure 1d; see the Supporting Information, section 4 for instrument setup and data analysis) extends from 1.14 ± 0.04 ns in NPLAs to 4.68 ± 0.11 ns in NPLEs, and the PLQY increases by 3.25 times, from 9.9 ± 0.2% in NPLAs to 32.3 ± 0.7% in NPLEs, consistent with literature reports.^33,35,69^ This suggests that passivation reduces the number of trap states in NPLE, although the PL measurement alone cannot differentiate between the passivation of electron or hole traps.^70^

Transient absorption (TA) measurements are conducted to investigate the effect of passivation on electron and hole dynamics. Experimental details can be found in the Supporting Information, section S5. The samples are excited at 400 nm at a low energy density (∼42 μJ/cm^2^ per pulse) to avoid multiexciton generation. The TA spectra of the NPLA sample at indicated delay times following excitation are shown in Figure 2a. The TA features have been assigned in our previous publication.^63^ Typically, the TA spectra are dominated by the exciton bleach (XB) at 451 nm contributed by both the band edge electron and hole^63,71,72^ and a broad featureless photoinduced absorption (PA) signal from 480 to 650 nm (inset in Figure 2a). Similar TA spectra are observed in NPLE, as shown in Figure S4. Figure 2b shows the XB kinetics in NPLA and NPLE. Both samples show a fast decay within 20 ps, while the NPLE XB decays slower at longer delay times (>100 ps). A power-dependent experiment (Supporting Information, section S7) verifies that the fast decay is an intrinsic process of the NPLs that occurs at single exciton conditions and cannot be attributed to the Auger recombination process of multiple exciton states.^73−75^ A similar fast decay component has been observed in other perovskite quantum dots and NPLs and was attributed to the fast electron trapping caused by halide vacancies.^55,76^ The slower XB decay (>100 ps) can be attributed to electron and hole recombination^71^ and/or slow carrier trapping processes.^70^ A slower XB decay in NPLE suggests that the addition of PbBr_2_ passivates electron and/or hole traps, decreasing their trapping rates. This is consistent with the PL decay results shown in Figure 1d. As will be discussed below, through selective ET and HT studies, it can be shown that this passivation procedure decreases electron traps, leading to longer PL lifetime and slower XB decay in NPLE.

**Figure 2:**
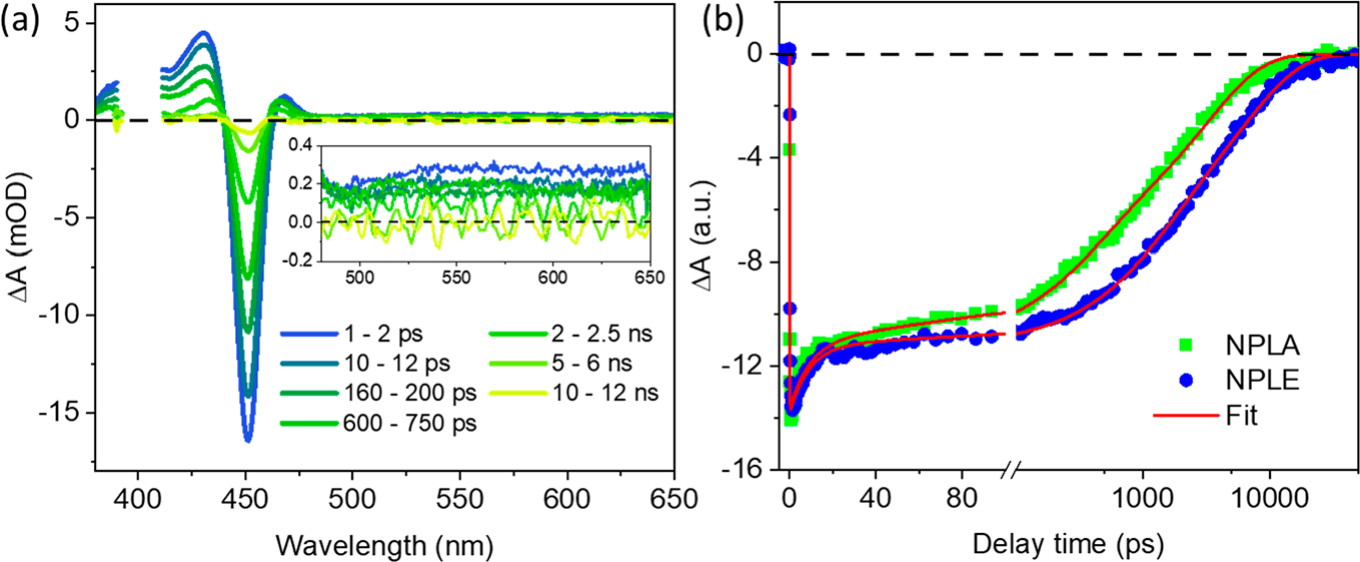
TA spectra and kinetics of NPL samples. (a) TA spectra of NPLAs at indicated delay times. Inset: a zoomed-in view of the spectra from 480 to 650 nm. (b) Comparison of normalized XB kinetics of NPLAs (green squares) and NPLEs (blue dots) and their fits (red lines).

To obtain further insight into the carrier dynamics that is responsible for the observed XB decay in NPLs (Figure 2), selective ET and HT from NPLs to electron and hole acceptors are also studied. For the ET experiments, methyl viologen dichloride (MV^2+^) and anthraquinone-2-carboxylic acid (AQA) are added to the surface of NPLAs to form NPLA-MV^2+^ and NPLA-AQA complexes, respectively. The absorption spectra of these complexes are shown in Figure S6a. The energy level alignment of the NPL CB and VB edge and the reduction potentials of electron acceptors are shown in Figure 3a and discussed in the Supporting Information, section S8. Details of the ET experiment and TA spectra and kinetics are given in the Supporting Information, section S9. The addition of MV^2+^ or AQA to NPLA solutions leads to a complete NPL PL quenching (Figure S6b), consistent with photoinduced ET from the excited NPLA to the electron acceptors, as expected from their energy level alignment. Direct evidence of ET is obtained from the TA results of NPLA-AQA shown in Figure 3b. Compared to pure NPLA, NPLA-AQA shows a broad positive peak centered at 600 nm (inset in Figure 3b), indicative of AQA^–^ generated by ET.^77,78^ In addition, this ET process is also confirmed by the ultrafast XB decay (Figure 3c) with a time constant of 0.49 ± 0.04 ps. Similarly, for NPLA-MV^2+^ complexes, the absorption of the ET product, MV^+•^ radicals, at ∼560 nm is observed in the TA spectra shown in Figure S6c, and a similar ultrafast ET induced XB bleach recovery with a time constant of 4.05 ± 0.31 ps can be seen in Figure 3d. It is important to note that these ultrafast ET processes are faster than the intrinsic XB decay in NPLAs without electron acceptors, outcompeting the carrier-trapping processes. Such ultrafast ET at the NPL surface may be facilitated by the strong electronic coupling with the electron and acceptor molecules enabled by the quantum confinement in NPLs.^8,79^ The fast ET also suggests that efficient extraction of excited electrons from the perovskite NPLs can be achieved in as-synthesized NPLs, without surface passivation treatment. Interestingly, in both NPLA-AQA and NPLA-MV^2+^ complexes, the XB contributed by the remaining hole in NPLs in the CS state (NPLA^+^-AQA^–^ and NPLA^+^-MV^+•^) decays with a time constant of tens of nanoseconds together with the reduced acceptors’ signal (Figure 3c,d and Table S2), indicative of the recombination of the transferred electron in the electron acceptor with the hole in the NPL. These XB bleach kinetics show negligible decay between tens of picoseconds and 1 ns, which suggests negligible trapping of the VB hole in as-synthesized NPLs in the <1 ns time scale. Thus, the XB decay within 1 ns in NPLA samples, shown in Figures 2b and 3c,d, cannot be attributed to a hole-trapping process in these materials. It is also worth noting that the electron–hole recombination times of the CS state in both NPLA-AQA and NPLA-MV^2+^ complexes are longer than that of intrinsic electron–hole recombination within NPLAs, which may be attributed to reduced electron–hole overlap in the CS state.

**Figure 3:**
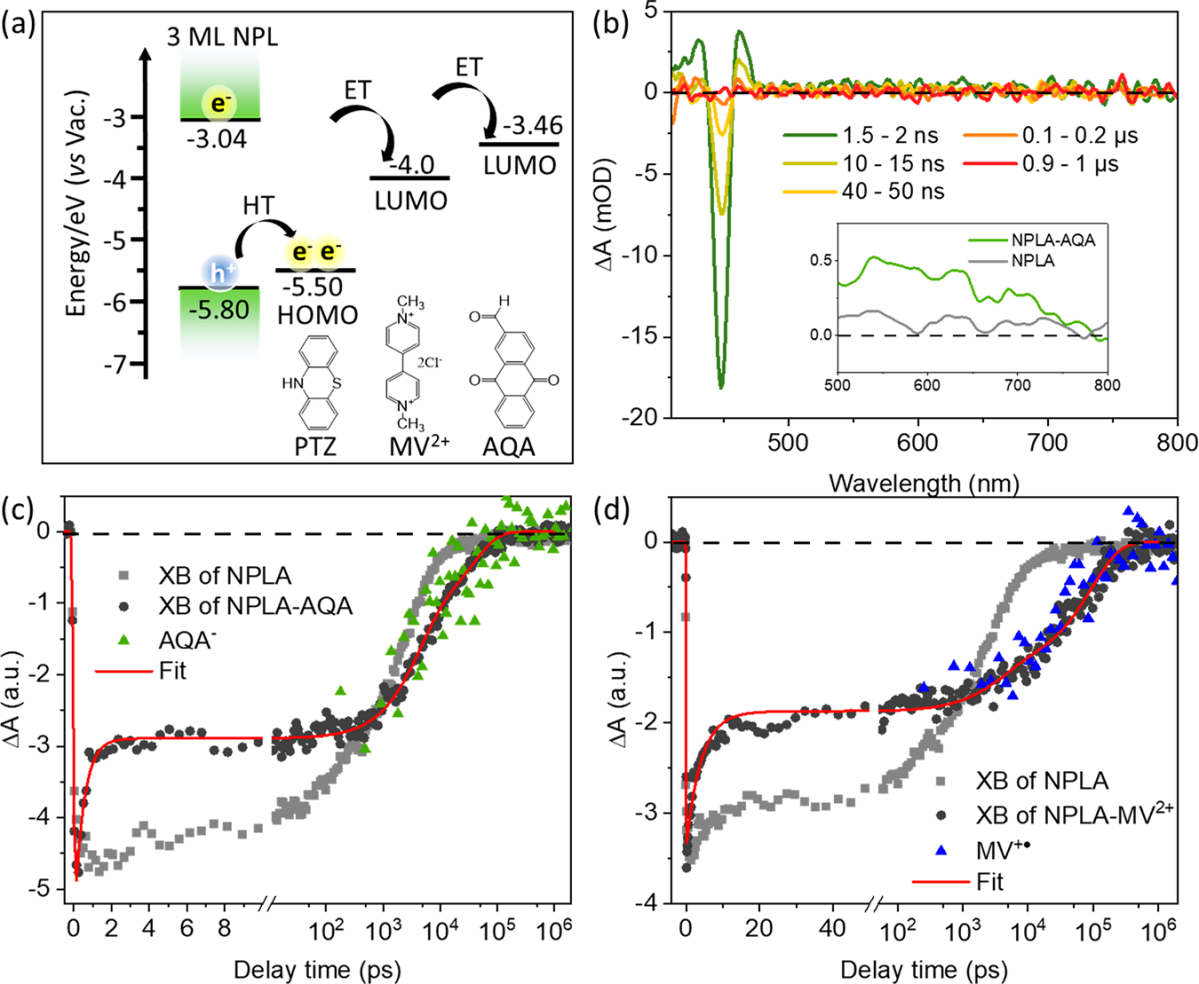
(a) Energy diagram of interfacial HT and ET from the 3 ML NPL to the highest occupied molecular orbital (HOMO) in phenothiazine (PTZ), the lowest unoccupied molecular orbital (LUMO) in methyl viologen dichloride (MV^2+^), and the LUMO in anthraquinone-2-carboxylic acid (AQA). Calculation of energy levels is discussed in the Supporting Information, section S8. (b) TA spectra of the NPLA-AQA complexes at indicated delay times after 400 nm excitation. Inset: a zoomed-in view of the TA spectrum of NPLA (gray line) and NPLA-AQA (green line) at 1.5–2 ns from 500 to 800 nm, showing the PA signal of reduced AQA (AQA^–^). (c and d) TA kinetics of the XB (black dots) and reduced electron acceptor (triangles) signals in NPLA-AQA and NPLA-MV^2+^, respectively. The XB kinetics of pure NPLA (gray squares) is also shown. The red lines represent multiexponential fits to the XB kinetics discussed in the Supporting Information, section S9.

For the HT experiment, PTZ is chosen as the hole acceptor for NPLA and NPLE, and the relevant energy levels are shown in Figures 3a and 4a (inset).^63^ Details of the sample preparation are given in the Supporting Information, section S10. The resulting complexes are denoted by NPLA-PTZ and NPLE-PTZ, respectively. Figure 4a shows that adding PTZ causes negligible changes to the ground-state absorption of NPLA (see Figure S7a for NPLE), while HT to PTZ causes ∼95% PL quenching, as quantified by the PL decay curves (Supporting Information, section S11). The TA spectra of NPLA-PTZ, shown in Figure 4b, consist of exciton band features similar to those of pure NPLA (Figure 2a) and an extra PA peak at 525 nm starting from ∼10 ps (inset in Figure 4b), which is attributed to the absorption of PTZ^+^ cation generated by HT.^63,80,81^ The formation and decay of the CS state (NPLA^–^-PTZ^+^) are monitored by the kinetics of the NPLA XB and PTZ^+^ PA signals, as shown in Figure 4c. Similar transient spectra and kinetics of NPLE-PTZ are shown in Figures S9a and 4d, respectively. In both NPLA-PTZ and NPLE-PTZ, the initial fast electron trapping is not affected by the presence of PTZ, as evidenced by the same decay kinetics within 10 ps between NPLA and NPLA-PTZ, or NPLE and NPLE-PTZ (Figure S9b,c). In addition, when compared to the XB kinetics of pure NPLA or NPLE, the XB in NPLA-PTZ or NPLE-PTZ shows a faster decay from 10 to 200 ps, together with the growth of the PTZ^+^ PA signal, confirming HT from the NPLA (or NPLE) to PTZ. With similar numbers of PTZ molecules per NPL in NPLA-PTZ and NPLE-PTZ (Supporting Information, section S10), the similar HT kinetics suggest that the surface passivation has negligible impact on HT.

**Figure 4:**
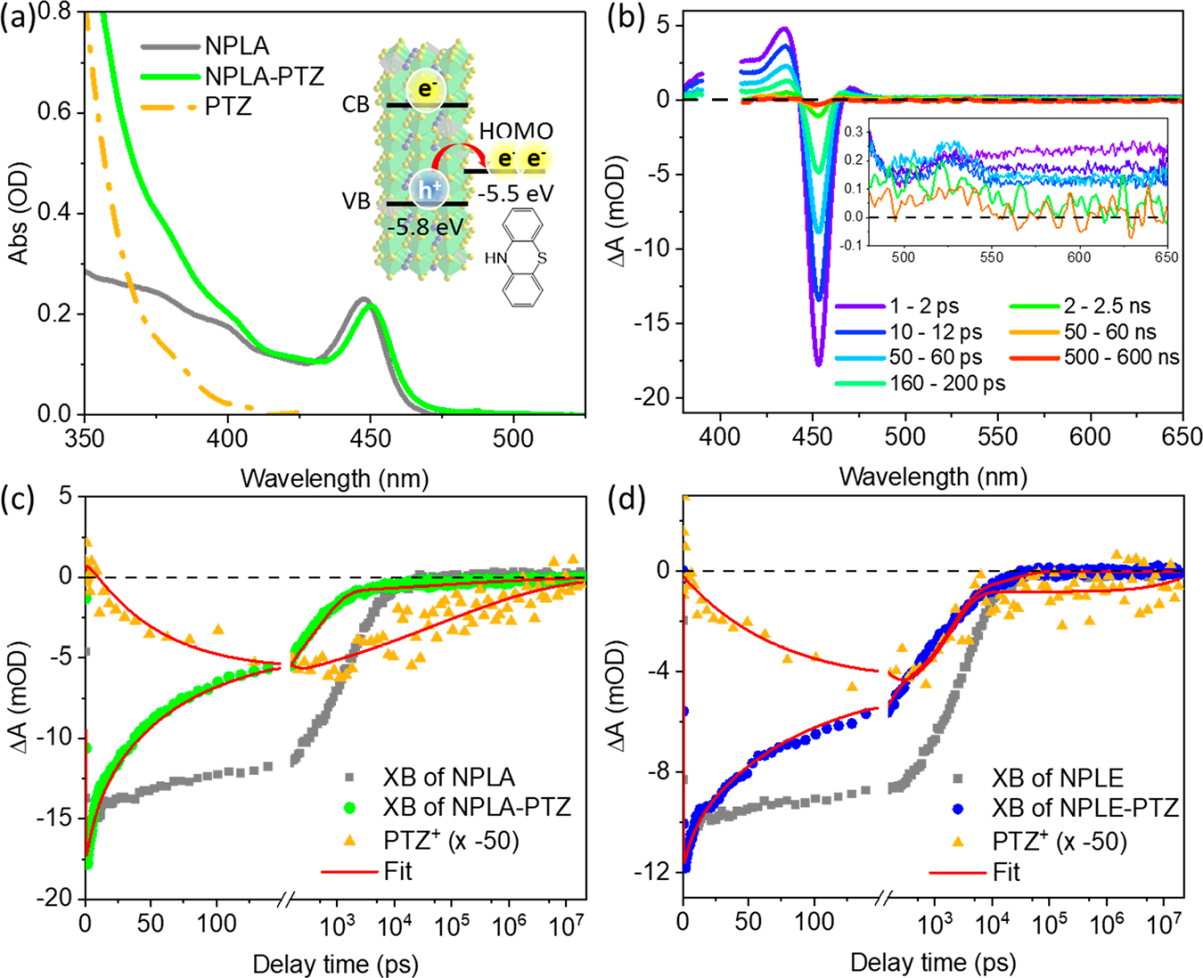
(a) Ground-state absorption spectra of NPLA and NPLA-PTZ. Subtraction of NPLA absorption from NPLA-PTZ generates the PTZ absorption (orange dash–dotted curve), as discussed in the Supporting Information, section S10. The inset is the schematic of HT from NPLA to PTZ. (b) TA spectra of NPLA-PTZ at indicated delay times. The inset shows the zoomed-in view from 480 to 650 nm. (c and d) Kinetics of XB (green and blue dots) and PTZ^+^ PA signal (orange triangles) in NPLA-PTZ and NPLE-PTZ, respectively. XB kinetics of pure NPLA and NPLE (gray squares) is shown for comparison. The kinetics of PTZ^+^ is flipped and scaled by 50 times for better comparison with XB kinetics. The red lines represent fitting results discussed in the Supporting Information, section S15.

The charge recombination (CR) process can be monitored by the decay of both the PTZ^+^ PA signal and the XB signal contributed by the electron remaining in NPLA or NPLE. In both NPLA-PTZ and NPLE-PTZ, after HT, the XB kinetics deviate from that of PTZ^+^, although the deviation is much smaller in the NPLE-PTZ sample. Specifically, in NPLA-PTZ (Figure 4c), after HT (>200 ps), the XB shows a two-phase decay behavior: most of the XB decays within 1 ns and a minor part decays on >100 ns scale, while the PTZ^+^ signal decays on the >100 ns time scale. For NPLE-PTZ, Figure 4d shows that most of the XB signal decays together with PTZ^+^ in 10 ns, indicating that most of the NPLE CB electrons recombine with the hole in PTZ^+^, consistent with the ET results discussed above and previous studies of charge separation and recombination at NC–acceptor interfaces.^71,77,82^ However, after the major CR, a minor part of the PTZ^+^ signal remains long-lived without a corresponding XB signal. These intriguing CR behaviors in NPLA-PTZ and NPLE-PTZ are confirmed by repeating the HT experiment with a new batch of NPLs, shown in the Supporting Information, section S12, Figure S10. As discussed in the Supporting Information, section S12, the observed significant discrepancy between the decay kinetics of XB and PTZ^+^ in NPLA-PTZ cannot be explained by sequential charge transfer to form the PTZ triplet.^83,84^ Instead, the CR kinetics difference between NPLA-PTZ and NPLE-PTZ suggests that the CB electron in NPLA-PTZ may decay into trap states after HT, and most of these electron trap states are passivated in the NPLE sample, as shown in Figure 5a,b.

**Figure 5:**
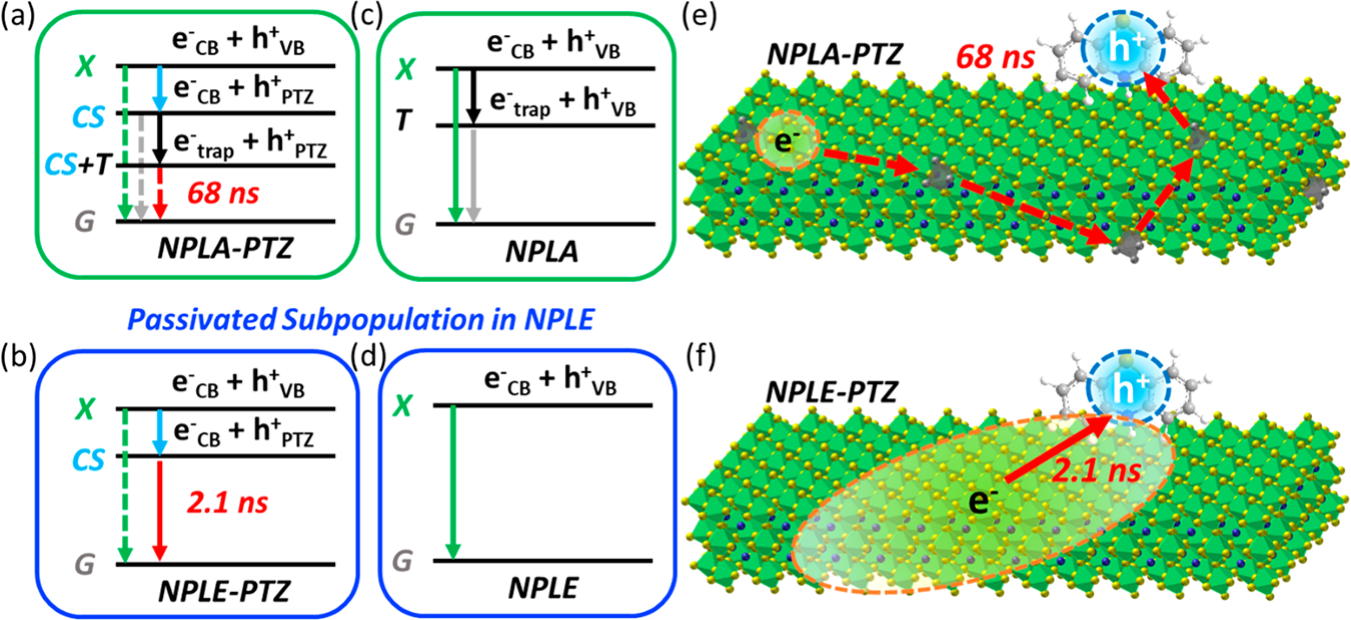
(a and b) Excited-state decay pathways in NPLA-PTZ and passivated NPLE-PTZ, respectively. In NPLA-PTZ, the exciton state (X), with electron in the CB (e^–^_CB_) and hole in the VB (h^+^_VB_), decays into the CS state (blue solid arrow), with the hole in PTZ (h^+^_PTZ_). The electron in the CS state then decays into a trap state (e^–^_trap_), forming the CS+T state, which finally decays to the ground state G. The dashed arrows indicate that the related decay pathway is relatively slow or outcompeted by other pathways. (c and d) Excited-state decay pathways in pure NPLA and passivated NPLE, respectively. (e and f) Illustration of the CS+T state in NPLA-PTZ and CS state in passivated NPLE-PTZ, respectively. The trapped electron in NPLA-PTZ is assumed to hop between Pb^2+^ vacancies (gray octahedrons) to recombine with the hole in PTZ.

The selective ET experimental results discussed above show that the VB hole in NPLAs is not trapped within 1 ns and that the fast XB decay in NPLA and NPLE can be attributed to electron trapping or electron–hole recombination. On the other hand, the HT results suggest that more electron traps are passivated in NPLE compared to NPLA. The excited-state decay pathways in NPLA and NPLE are summarized in panels c and d of Figure 5, respectively. Thus, the XB kinetics in NPLA and NPLE (Figure 2b), in the absence of electron or hole acceptors, is fit by eq 1.
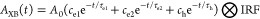
1In eq 1, *A*_0_ is the maximum amplitude of XB signals, *c*_e_ (*c*_h_) is the electron (hole) contribution to the XB. τ_e1_ is the initial fast electron trapping lifetime observed in both NPLA and NPLE (Figure 2b). τ_e2_ is the passivation-dependent electron decay lifetime. In NPLA, τ_e2_ is contributed by electron–hole recombination to the ground state and extra electron trapping, represented by the green and black arrows in Figure 5c, respectively. On the other hand, this electron-trapping step is passivated in NPLE, as shown in Figure 5d. Note that the first electron decay pathway (τ_e1_) is not shown in Figure 5 for simplicity since it shows no dependence on the surface passivation studied here or has no impact on the interfacial charge transfer kinetics (Figures 3, 4, and S10). Also, Figure 5b,d shows only the passivated subpopulation in the NPLE sample. As discussed below, a minor portion of the NPLE sample is not passivated and behaves as NPLA, as shown in Figure 5a,c. τ_h_ represents the hole recombination lifetime with the electron, as no hole trapping was observed in the ET experiments. The XB kinetics of NPLA and NPLE are globally fit to eq 1 with shared *c*_e1_, *c*_e2_, *c*_h_, and τ_e1_. More details of the fitting are provided in Supporting Information, section S13. The fitting yields an electron (hole) contribution of 48.3 ± 1.3% (51.7 ± 1.3%), close to the theoretical value (50%) obtained by analyzing the NPL band edge fine structures (Supporting Information, section S14).^85−88^ The fit reveals τ_e1_ of 9.0 ± 0.6 ps in both NPLA and NPLE and τ_e2_ of 356.4 ± 27.1 and 1150 ± 75 ps for NPLA and NPLE, respectively. The difference in τ_e2_ is attributed to additional electron trapping in NPLA which is passivated in NPLE. As discussed in the Supporting Information, section S13, the lifetime of this extra electron trapping is calculated according to eq 2.^48,70^
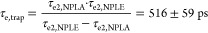
2

As discussed above, the fast electron trap (τ_e1_ = 9.0 ± 0.6 ps) is due to Br^–^ vacancies which are not passivated by PbBr_2_. The possible reason is that the Br:Pb ratio cannot be elevated to 10:3 by excess PbBr_2_ assuming a uniform distribution of the passivation species on the NPL surface. An extra Br^–^ source may be necessary to further passivate the NPLs. The 516 ± 59 ps electron trapping is also consistent with the literature.^33,35,38^ Bohn et al. showed that, when increasing the passivation extent, a 50–500 ps trapping component in the TA kinetics of the same NPLs gradually decreases.^33^ Similarly, the TA data reported by Wu et al. shows a hundreds of picosecond difference after PbBr_6_^4–^ octahedra passivation.^35^ These slow electron traps can be passivated by PbBr_2_, suggesting that the corresponding electronic state may be caused by Pb^2+^ vacancies. Previous simulations suggest that a shortened Pb–Pb distance in 2D lead halide perovskite creates electronic states below the CB minimum, favoring the formation of a trapped electron or a small electron polaron.^89^ From the passivation results observed in this work, it is speculated that the appearance of Pb^2+^ vacancies in NPLAs renders a more deformable crystal structure and thus a higher possibility of finding shortened Pb–Pb distances, or small electron polarons, surrounding the Pb^2+^ vacancies in the excited state. Time-resolved structural studies^90,91^ and computational modeling may help to reveal such slow electron-trapping process; however, this is beyond the scope of this work.

With the electron trapping lifetimes (τ_e1_ and τ_e,trap_) extracted from pure NPLs, the XB kinetics of NPLA-PTZ and NPLE-PTZ (Figure 4c,d) are fitted with and without τ_e,trap_, respectively, through a multiexponential decay function according to the model in Figure 5a,b. The fitting details and parameters are provided in the Supporting Information, section S15. As shown in Figure 4c,d, the XB kinetics are fit well for both NPLA-PTZ and NPLE-PTZ. The fitting reveals an HT time constant of 60.0 ± 5.4 and 78.4 ± 8.1 ps in NPLA-PTZ and NPLE-PTZ, respectively, confirming that the passivation of electron traps has negligible impact on hole extraction. On the other hand, the CR lifetime exhibits dramatic dependence on electron trap passivation. As shown in Figure 4c,d, the CR kinetics, monitored by the PTZ^+^ PA signal, can be globally fit with the XB kinetics (Supporting Information, section S15). In NPLA-PTZ, the PTZ^+^ PA signal decay is fit by a stretched exponential function (eq S9), which yields a CR half-lifetime of 68 ± 3 ns, consistent with our previous report (>100 ns).^63^ Similar long CR lifetimes have been observed in many other perovskite NC-molecular charge acceptor complexes.^77,92,93^ However, after surface passivation, the CR half-lifetime is reduced to 2.1 ± 0.1 ns in NPLE-PTZ (Figure 4d).

The longer CR lifetime with trapped electron in NPLA-PTZ may be understood by considering the spatial separation between the electron and hole.^43^ As shown in Figure 5e, the transferred hole is localized in one PTZ molecule on the NPL surface, while the electron decays into the trap state around Pb^2+^ vacancies, decreasing the wave function overlap between the electron and hole and extending the lifetime of the CS state. The distance between the electron and hole depends on the NPL size and the density of Pb^2+^ vacancies or trap states. A heterogeneous distribution of the electron–hole distance in the NPLA-PTZ ensemble may explain the necessity of using a stretched exponential function to fit the CR kinetics. Note that the XB in NPLA-PTZ shows a minor long-lived amplitude (>100 ns, Figure S13a), which can be explained by an electron trapping–detrapping model with a trap depth of 36–46 meV (Supporting Information, sections S16 and S17) and may imply that the trapped electrons can hop between Pb^2+^ vacancies before final recombination with PTZ^+^ (Figures 5e and S13c), similar to the trapped carrier motion observed in bulk 2D perovskites^94^ and CdS NRs.^44^ An NPL size-dependent or trap density-dependent HT experiment may offer more insights into the motion of the trapped electron in the CS state. The trapping-induced fast XB decay (516 ps) after HT was not observed in the aforementioned studies where long CR lifetime was measured in cuboidal perovskite NCs.^77,92,93^ This may indicate that the formation of trapped electrons is unique to the perovskite NPLs and may be enabled by the 2D morphology and the large surface-to-volume ratio.

In NPLE-PTZ, however, with a reduced amount of electron traps, as shown in Figure 5f, the fast recombination is facilitated by the increased electron–hole wave function overlap between the strongly confined CB electron in NPLE and the oxidized acceptor.^63,71,95^ The remaining long-lived PTZ^+^ PA signal (16%; Supporting Information, sections S15 and S16) in NPLE-PTZ (Figure 4d) may be due to the incomplete passivation of NPLs in the ensemble, which undergoes the same recombination as in NPLA-PTZ and shows long lifetime. The remaining 16% of the slow electron traps as well as the fast trapping process that cannot be removed by our passivation procedure (see above) are responsible for the nonunity PLQY (32.3 ± 0.7%) of the NPLE samples.

The above charge transfer experiments demonstrate the tunability of the electron dynamics in perovskite NPLs through surface PbBr_2_ passivation, which offers more possibilities for the application of these NPLs. For light emitting applications, passivation of electron traps ensures a high PL quantum yield and therefore a better device performance.^33,35^ Our study here suggests that in addition to PbBr_2_, an extra Br^–^ source may be necessary to further improve the NPL PL efficiency. On the other hand, the presence of electron traps introduces negligible impact on HT but enables a long-lived CS state, while efficient ET to selected acceptors can still outcompete electron trapping, benefiting their applications in photovoltaics and photocatalysis.

To summarize, the surface passivation of 3 ML L_2_Cs_*n*–1_Pb_*n*_Br_3*n*+1_ NPLs and its influence on charge transfer are studied by ultrafast spectroscopies. Two types of electron traps with distinct trapping lifetimes of 9.0 ± 0.6 and 516 ± 59 ps are revealed by transient absorption studies, while negligible hole traps are present in the NPLs. Through studies of charge transfer to electron and hole acceptors, we show that electron traps have negligible impact on either electron or hole transfer but enable a long-lived CS state after hole transfer, which is attributed to the reduced electron–hole wave function overlap. This work unveils the carrier trap identity and its influence on charge transfer in 2D lead halide perovskite NPLs, offering guidelines for further improving their applications in light-emitting devices, photovoltaics, and photocatalysis.

## Experimental Methods

### Sample Preparation

The perovskite NPL synthesis and surface passivation were conducted following a previous report^33^ with slight modifications. NPL-acceptor complexes were prepared by sonicating excessive acceptor powder in NPL colloids. Details can be found in the Supporting Information, section S1.

### Experimental Setup

Transient absorption measurements were based on a regenerative amplified Ti:Sapphire femtosecond laser system (Astrella, Coherent, 800 nm, 1 kHz, 35 fs pulse duration, and 5.1 mJ/pulse). Time-resolved PL decay was measured using a time-correlated single-photon-counting technique (Becker & Hickel SPC 600). More details of instrumentation for optical characterizations can be found in the Supporting Information, sections S2, S4, and S5.

## Supporting Information Available

Sample preparation, TEM characterization, experiment setups, PL and TA spectra, kinetics derivation, and fittings (PDF)

## References

[ref1] Schmidt L. C.; Pertegas A.; Gonzalez-Carrero S.; Malinkiewicz O.; Agouram S.; Minguez Espallargas G.; Bolink H. J.; Galian R. E.; Perez-Prieto J. Nontemplate Synthesis of CH3NH3PbBr3 Perovskite Nanoparticles. J. Am. Chem. Soc. 2014, 136 (3), 850–853. 10.1021/ja4109209. 24387158

[ref2] Protesescu L.; Yakunin S.; Bodnarchuk M. I.; Krieg F.; Caputo R.; Hendon C. H.; Yang R. X.; Walsh A.; Kovalenko M. V. Nanocrystals of Cesium Lead Halide Perovskites (CsPbX(3), X = Cl, Br, and I): Novel Optoelectronic Materials Showing Bright Emission with Wide Color Gamut. Nano Lett. 2015, 15 (6), 3692–3696. 10.1021/nl5048779. 25633588 PMC4462997

[ref3] Miyata K.; Atallah T. L.; Zhu X. Y. Lead Halide Perovskites: Crystal-Liquid Duality, Phonon Glass Electron Crystals, and Large Polaron Formation. Sci. Adv. 2017, 3 (10), 1701469 10.1126/sciadv.1701469. PMC5640380 29043296

[ref4] Martiradonna L. Riddles in Perovskite Research. Nat. Mater. 2018, 17 (5), 377 10.1038/s41563-018-0072-y. 29686242

[ref5] Zhu X. Y.; Podzorov V. Charge Carriers in Hybrid Organic-Inorganic Lead Halide Perovskites Might Be Protected as Large Polarons. J. Phys. Chem. Lett. 2015, 6 (23), 4758–4761. 10.1021/acs.jpclett.5b02462. 26575427

[ref6] Dey A.; Ye J.; De A.; Debroye E.; Ha S. K.; Bladt E.; Kshirsagar A. S.; Wang Z.; Yin J.; Wang Y.; et al. State of the Art and Prospects for Halide Perovskite Nanocrystals. ACS Nano 2021, 15 (7), 10775–10981. 10.1021/acsnano.0c08903. 34137264 PMC8482768

[ref7] Shamsi J.; Urban A. S.; Imran M.; De Trizio L.; Manna L. Metal Halide Perovskite Nanocrystals: Synthesis, Post-Synthesis Modifications, and Their Optical Properties. Chem. Rev. 2019, 119 (5), 3296–3348. 10.1021/acs.chemrev.8b00644. 30758194 PMC6418875

[ref8] Katan C.; Mercier N.; Even J. Quantum and Dielectric Confinement Effects in Lower-Dimensional Hybrid Perovskite Semiconductors. Chem. Rev. 2019, 119 (5), 3140–3192. 10.1021/acs.chemrev.8b00417. 30638375

[ref9] Akkerman Q. A.; D’Innocenzo V.; Accornero S.; Scarpellini A.; Petrozza A.; Prato M.; Manna L. Tuning the Optical Properties of Cesium Lead Halide Perovskite Nanocrystals by Anion Exchange Reactions. J. Am. Chem. Soc. 2015, 137 (32), 10276–10281. 10.1021/jacs.5b05602. 26214734 PMC4543997

[ref10] Nedelcu G.; Protesescu L.; Yakunin S.; Bodnarchuk M. I.; Grotevent M. J.; Kovalenko M. V. Fast Anion-Exchange in Highly Luminescent Nanocrystals of Cesium Lead Halide Perovskites (CsPbX3, X = Cl, Br, I). Nano Lett. 2015, 15 (8), 5635–5640. 10.1021/acs.nanolett.5b02404. 26207728 PMC4538456

[ref11] Jang D. M.; Park K.; Kim D. H.; Park J.; Shojaei F.; Kang H. S.; Ahn J. P.; Lee J. W.; Song J. K. Reversible Halide Exchange Reaction of Organometal Trihalide Perovskite Colloidal Nanocrystals for Full-Range Band Gap Tuning. Nano Lett. 2015, 15 (8), 5191–5199. 10.1021/acs.nanolett.5b01430. 26161637

[ref12] Ye J.; Byranvand M. M.; Martinez C. O.; Hoye R. L. Z.; Saliba M.; Polavarapu L. Defect Passivation in Lead-Halide Perovskite Nanocrystals and Thin Films: Toward Efficient LEDs and Solar Cells. Angew. Chem. Int. Ed 2021, 60 (40), 21636–21660. 10.1002/anie.202102360. PMC8518834 33730428

[ref13] Yin J.; Yang H.; Gutierrez-Arzaluz L.; Zhou Y.; Bredas J. L.; Bakr O. M.; Mohammed O. F. Luminescence and Stability Enhancement of Inorganic Perovskite Nanocrystals via Selective Surface Ligand Binding. ACS Nano 2021, 15 (11), 17998–18005. 10.1021/acsnano.1c06480. 34723469

[ref14] Nenon D. P.; Pressler K.; Kang J.; Koscher B. A.; Olshansky J. H.; Osowiecki W. T.; Koc M. A.; Wang L. W.; Alivisatos A. P. Design Principles for Trap-Free CsPbX3 Nanocrystals: Enumerating and Eliminating Surface Halide Vacancies with Softer Lewis Bases. J. Am. Chem. Soc. 2018, 140 (50), 17760–17772. 10.1021/jacs.8b11035. 30501174

[ref15] Krieg F.; Ochsenbein S. T.; Yakunin S.; Ten Brinck S.; Aellen P.; Süess A.; Clerc B.; Guggisberg D.; Nazarenko O.; Shynkarenko Y.; et al. Colloidal CsPbX3 (X = Cl, Br, I) Nanocrystals 2.0: Zwitterionic Capping Ligands for Improved Durability and Stability. ACS Energy Lett. 2018, 3 (3), 641–646. 10.1021/acsenergylett.8b00035. 29552638 PMC5848145

[ref16] He Y.; Liang Y.; Liang S.; Harn Y. W.; Li Z.; Zhang M.; Shen D.; Li Z.; Yan Y.; Pang X.; et al. Dual-Protected Metal Halide Perovskite Nanosheets with an Enhanced Set of Stabilities. Angew. Chem. Int. Ed 2021, 133 (13), 7335–7342. 10.1002/ange.202014983. 33393190

[ref17] DuBose J. T.; Kamat P. V. Surface Chemistry Matters. How Ligands Influence Excited State Interactions between CsPbBr3 and Methyl Viologen. J. Phys. Chem. C 2020, 124 (24), 12990–12998. 10.1021/acs.jpcc.0c03004.

[ref18] Kim Y. H.; Zhai Y.; Lu H.; Pan X.; Xiao C.; Gaulding E. A.; Harvey S. P.; Berry J. J.; Vardeny Z. V.; Luther J. M.; et al. Chiral-Induced Spin Selectivity Enables a Room-Temperature Spin Light-Emitting Diode. Science 2021, 371 (6534), 1129–1133. 10.1126/science.abf5291. 33707260

[ref19] Dong Y.; Wang Y. K.; Yuan F.; Johnston A.; Liu Y.; Ma D.; Choi M. J.; Chen B.; Chekini M.; Baek S. W.; et al. Bipolar-Shell Resurfacing for Blue LEDs Based on Strongly Confined Perovskite Quantum Dots. Nat. Nanotechnol 2020, 15 (8), 668–674. 10.1038/s41565-020-0714-5. 32632321

[ref20] Kim Y. H.; Kim S.; Kakekhani A.; Park J.; Park J.; Lee Y. H.; Xu H. X.; Nagane S.; Wexler R. B.; Kim D. H.; et al. Comprehensive Defect Suppression in Perovskite Nanocrystals for High-Efficiency Light-Emitting Diodes. Nat. Photonics 2021, 15 (2), 148–155. 10.1038/s41566-020-00732-4.

[ref21] Swarnkar A.; Marshall A. R.; Sanehira E. M.; Chernomordik B. D.; Moore D. T.; Christians J. A.; Chakrabarti T.; Luther J. M. Quantum Dot-Induced Phase Stabilization of Alpha-CsPbI3 Perovskite for High-Efficiency Photovoltaics. Science 2016, 354 (6308), 92–95. 10.1126/science.aag2700. 27846497

[ref22] Hao M.; Bai Y.; Zeiske S.; Ren L.; Liu J.; Yuan Y.; Zarrabi N.; Cheng N.; Ghasemi M.; Chen P.; et al. Ligand-Assisted Cation-Exchange Engineering for High-Efficiency Colloidal Cs1–xFAxPbI3 Quantum Dot Solar Cells with Reduced Phase Segregation. Nat. Energy 2020, 5 (1), 79–88. 10.1038/s41560-019-0535-7.

[ref23] Li L.; Zhang Z. J.; Ding C.; Xu J. Y. Boosting Charge Separation and Photocatalytic CO2 Reduction of CsPbBr3 Perovskite Quantum Dots by Hybridizing with P3HT. Chem. Eng. J. 2021, 419, 129543 10.1016/j.cej.2021.129543.

[ref24] Xu Y. F.; Yang M. Z.; Chen B. X.; Wang X. D.; Chen H. Y.; Kuang D. B.; Su C. Y. A CsPbBr3 Perovskite Quantum Dot/Graphene Oxide Composite for Photocatalytic CO2 Reduction. J. Am. Chem. Soc. 2017, 139 (16), 5660–5663. 10.1021/jacs.7b00489. 28385017

[ref25] Wang H.; Wang X.; Chen R.; Zhang H.; Wang X.; Wang J.; Zhang J.; Mu L.; Wu K.; Fan F.; et al. Promoting Photocatalytic H2 Evolution on Organic–Inorganic Hybrid Perovskite Nanocrystals by Simultaneous Dual-Charge Transportation Modulation. ACS Energy Lett. 2019, 4 (1), 40–47. 10.1021/acsenergylett.8b01830.

[ref26] Zhu X.; Lin Y.; Sun Y.; Beard M. C.; Yan Y. Lead-Halide Perovskites for Photocatalytic alpha-Alkylation of Aldehydes. J. Am. Chem. Soc. 2019, 141 (2), 733–738. 10.1021/jacs.8b08720. 30602111

[ref27] Schanze K. S.; Kamat P. V.; Yang P.; Bisquert J. Progress in Perovskite Photocatalysis. ACS Energy Lett. 2020, 5 (8), 2602–2604. 10.1021/acsenergylett.0c01480.

[ref28] Wang K.; Lu H.; Zhu X.; Lin Y.; Beard M. C.; Yan Y.; Chen X. Ultrafast Reaction Mechanisms in Perovskite Based Photocatalytic C–C Coupling. ACS Energy Lett. 2020, 5 (2), 566–571. 10.1021/acsenergylett.9b02714.

[ref29] Otero-Martinez C.; Ye J.; Sung J.; Pastoriza-Santos I.; Perez-Juste J.; Xia Z.; Rao A.; Hoye R. L. Z.; Polavarapu L. Colloidal Metal-Halide Perovskite Nanoplatelets: Thickness-Controlled Synthesis, Properties, and Application in Light-Emitting Diodes. Adv. Mater. 2022, 34 (10), 2107105 10.1002/adma.202107105. 34775643

[ref30] Ha S. K.; Mauck C. M.; Tisdale W. A. Toward Stable Deep-Blue Luminescent Colloidal Lead Halide Perovskite Nanoplatelets: Systematic Photostability Investigation. Chem. Mater. 2019, 31 (7), 2486–2496. 10.1021/acs.chemmater.8b05310.

[ref31] Weidman M. C.; Goodman A. J.; Tisdale W. A. Colloidal Halide Perovskite Nanoplatelets: An Exciting New Class of Semiconductor Nanomaterials. Chem. Mater. 2017, 29 (12), 5019–5030. 10.1021/acs.chemmater.7b01384.

[ref32] Weidman M. C.; Seitz M.; Stranks S. D.; Tisdale W. A. Highly Tunable Colloidal Perovskite Nanoplatelets through Variable Cation, Metal, and Halide Composition. ACS Nano 2016, 10 (8), 7830–7839. 10.1021/acsnano.6b03496. 27471862

[ref33] Bohn B. J.; Tong Y.; Gramlich M.; Lai M. L.; Doblinger M.; Wang K.; Hoye R. L. Z.; Muller-Buschbaum P.; Stranks S. D.; Urban A. S.; et al. Boosting Tunable Blue Luminescence of Halide Perovskite Nanoplatelets through Postsynthetic Surface Trap Repair. Nano Lett. 2018, 18 (8), 5231–5238. 10.1021/acs.nanolett.8b02190. 29990435

[ref34] Polavarapu L.; Nickel B.; Feldmann J.; Urban A. S. Advances in Quantum-Confined Perovskite Nanocrystals for Optoelectronics. Adv. Energy Mater. 2017, 7 (16), 1700267 10.1002/aenm.201700267.

[ref35] Wu Y.; Wei C.; Li X.; Li Y.; Qiu S.; Shen W.; Cai B.; Sun Z.; Yang D.; Deng Z.; et al. In Situ Passivation of PbBr64– Octahedra toward Blue Luminescent CsPbBr3 Nanoplatelets with Near 100% Absolute Quantum Yield. ACS Energy Lett. 2018, 3 (9), 2030–2037. 10.1021/acsenergylett.8b01025.

[ref36] Bertolotti F.; Nedelcu G.; Vivani A.; Cervellino A.; Masciocchi N.; Guagliardi A.; Kovalenko M. V. Crystal Structure, Morphology, and Surface Termination of Cyan-Emissive, Six-Monolayers-Thick CsPbBr3 Nanoplatelets from X-ray Total Scattering. ACS Nano 2019, 13 (12), 14294–14307. 10.1021/acsnano.9b07626. 31747248 PMC6933817

[ref37] Hoye R. L. Z.; Lai M. L.; Anaya M.; Tong Y.; Galkowski K.; Doherty T.; Li W.; Huq T. N.; Mackowski S.; Polavarapu L.; et al. Identifying and Reducing Interfacial Losses to Enhance Color-Pure Electroluminescence in Blue-Emitting Perovskite Nanoplatelet Light-Emitting Diodes. ACS Energy Lett. 2019, 4 (5), 1181–1188. 10.1021/acsenergylett.9b00571. 31119197 PMC6516044

[ref38] Vonk S. J. W.; Fridriksson M. B.; Hinterding S. O. M.; Mangnus M. J. J.; van Swieten T. P.; Grozema F. C.; Rabouw F. T.; van der Stam W. Trapping and Detrapping in Colloidal Perovskite Nanoplatelets: Elucidation and Prevention of Nonradiative Processes through Chemical Treatment. J. Phys. Chem. C 2020, 124 (14), 8047–8054. 10.1021/acs.jpcc.0c02287. PMC7217613 32421082

[ref39] Gao Y.; Peng X. Photogenerated Excitons in Plain Core CdSe Nanocrystals with Unity Radiative Decay in Single Channel: the Effects of Surface and Ligands. J. Am. Chem. Soc. 2015, 137 (12), 4230–4235. 10.1021/jacs.5b01314. 25785631

[ref40] Pu C.; Peng X. To Battle Surface Traps on CdSe/CdS Core/Shell Nanocrystals: Shell Isolation versus Surface Treatment. J. Am. Chem. Soc. 2016, 138 (26), 8134–8142. 10.1021/jacs.6b02909. 27312799

[ref41] Wu K.; Zhu H.; Liu Z.; Rodriguez-Cordoba W.; Lian T. Ultrafast Charge Separation and Long-Lived Charge Separated State in Photocatalytic CdS-Pt Nanorod Heterostructures. J. Am. Chem. Soc. 2012, 134 (25), 10337–10340. 10.1021/ja303306u. 22655858

[ref42] Wu K.; Li Q.; Du Y.; Chen Z.; Lian T. Ultrafast Exciton Quenching by Energy and Electron Transfer in Colloidal CdSe Nanosheet-Pt Heterostructures. Chem. Sci. 2015, 6 (2), 1049–1054. 10.1039/C4SC02994A. 29560193 PMC5811111

[ref43] Li Q.; Zhao F.; Qu C.; Shang Q.; Xu Z.; Yu L.; McBride J. R.; Lian T. Two-Dimensional Morphology Enhances Light-Driven H2 Generation Efficiency in CdS Nanoplatelet-Pt Heterostructures. J. Am. Chem. Soc. 2018, 140 (37), 11726–11734. 10.1021/jacs.8b06100. 30145886

[ref44] Utterback J. K.; Grennell A. N.; Wilker M. B.; Pearce O. M.; Eaves J. D.; Dukovic G. Observation of Trapped-Hole Diffusion on the Surfaces of CdS Nanorods. Nat. Chem. 2016, 8 (11), 1061–1066. 10.1038/nchem.2566. 27768112

[ref45] Tarafder K.; Surendranath Y.; Olshansky J. H.; Alivisatos A. P.; Wang L. W. Hole Transfer Dynamics From a CdSe/CdS Quantum Rod to a Tethered Ferrocene Derivative. J. Am. Chem. Soc. 2014, 136 (13), 5121–5131. 10.1021/ja500936n. 24654595

[ref46] Olshansky J. H.; Balan A. D.; Ding T. X.; Fu X.; Lee Y. V.; Alivisatos A. P. Temperature-Dependent Hole Transfer from Photoexcited Quantum Dots to Molecular Species: Evidence for Trap-Mediated Transfer. ACS Nano 2017, 11 (8), 8346–8355. 10.1021/acsnano.7b03580. 28759718

[ref47] Yan C.; Weinberg D.; Jasrasaria D.; Kolaczkowski M. A.; Liu Z. J.; Philbin J. P.; Balan A. D.; Liu Y.; Schwartzberg A. M.; Rabani E.; et al. Uncovering the Role of Hole Traps in Promoting Hole Transfer from Multiexcitonic Quantum Dots to Molecular Acceptors. ACS Nano 2021, 15 (2), 2281–2291. 10.1021/acsnano.0c08158. 33336575

[ref48] Jin T.; Lian T. Trap State Mediated Triplet Energy Transfer from CdSe Quantum Dots to Molecular Acceptors. J. Chem. Phys. 2020, 153 (7), 074703 10.1063/5.0022061. 32828113

[ref49] Rigsby E. M.; Lee K.; Sun J.; Fishman D. A.; Tang M. L. Primary Amines Enhance Triplet Energy Transfer from Both the Band Edge and Trap State from CdSe Nanocrystals. J. Chem. Phys. 2019, 151 (17), 174701 10.1063/1.5125021. 31703522

[ref50] Han Y.; He S.; Luo X.; Li Y.; Chen Z.; Kang W.; Wang X.; Wu K. Triplet Sensitization by ″Self-Trapped″ Excitons of Nontoxic CuInS2 Nanocrystals for Efficient Photon Upconversion. J. Am. Chem. Soc. 2019, 141 (33), 13033–13037. 10.1021/jacs.9b07033. 31393119

[ref51] Bender J. A.; Raulerson E. K.; Li X.; Goldzak T.; Xia P.; Van Voorhis T.; Tang M. L.; Roberts S. T. Surface States Mediate Triplet Energy Transfer in Nanocrystal-Acene Composite Systems. J. Am. Chem. Soc. 2018, 140 (24), 7543–7553. 10.1021/jacs.8b01966. 29846066

[ref52] Huang H.; Bodnarchuk M. I.; Kershaw S. V.; Kovalenko M. V.; Rogach A. L. Lead Halide Perovskite Nanocrystals in the Research Spotlight: Stability and Defect Tolerance. ACS Energy Lett. 2017, 2 (9), 2071–2083. 10.1021/acsenergylett.7b00547. 28920080 PMC5594444

[ref53] Kovalenko M. V.; Protesescu L.; Bodnarchuk M. I. Properties and Potential Optoelectronic Applications of Lead Halide Perovskite Nanocrystals. Science 2017, 358 (6364), 745–750. 10.1126/science.aam7093. 29123061

[ref54] Akkerman Q. A.; Raino G.; Kovalenko M. V.; Manna L. Genesis, Challenges and Opportunities for Colloidal Lead Halide Perovskite Nanocrystals. Nat. Mater. 2018, 17 (5), 394–405. 10.1038/s41563-018-0018-4. 29459748

[ref55] Lai R.; Wu K. Picosecond Electron Trapping Limits the Emissivity of CsPbCl3 Perovskite Nanocrystals. J. Chem. Phys. 2019, 151 (19), 194701 10.1063/1.5127887. 31757160

[ref56] Mondal N.; De A.; Samanta A. Achieving Near-Unity Photoluminescence Efficiency for Blue-Violet-Emitting Perovskite Nanocrystals. ACS Energy Lett. 2019, 4 (1), 32–39. 10.1021/acsenergylett.8b01909.

[ref57] Buin A.; Comin R.; Xu J.; Ip A. H.; Sargent E. H. Halide-Dependent Electronic Structure of Organolead Perovskite Materials. Chem. Mater. 2015, 27 (12), 4405–4412. 10.1021/acs.chemmater.5b01909.

[ref58] Liu X. C.; Zeng P.; Chen S. H.; Smith T. A.; Liu M. Z. Charge Transfer Dynamics at the Interface of CsPbX3 Perovskite Nanocrystal-Acceptor Complexes: A Femtosecond Transient Absorption Spectroscopy Study. Laser Photonics Rev. 2022, 16 (12), 2200280 10.1002/lpor.202200280.

[ref59] Palabathuni M.; Akhil S.; Singh R.; Mishra N. Charge Transfer in Photoexcited Cesium-Lead Halide Perovskite Nanocrystals: Review of Materials and Applications. ACS Appl. Nano Mater. 2022, 5 (8), 10097–10117. 10.1021/acsanm.2c01550.

[ref60] DuBose J. T.; Kamat P. V. Energy Versus Electron Transfer: Managing Excited-State Interactions in Perovskite Nanocrystal-Molecular Hybrids. Chem. Rev. 2022, 122 (15), 12475–12494. 10.1021/acs.chemrev.2c00172. 35793168

[ref61] Mondal N.; De A.; Das S.; Paul S.; Samanta A. Ultrafast Carrier Dynamics of Metal Halide Perovskite Nanocrystals and Perovskite-Composites. Nanoscale 2019, 11 (20), 9796–9818. 10.1039/C9NR01745C. 31070653

[ref62] Gramlich M.; Lampe C.; Drewniok J.; Urban A. S. How Exciton-Phonon Coupling Impacts Photoluminescence in Halide Perovskite Nanoplatelets. J. Phys. Chem. Lett. 2021, 12 (46), 11371–11377. 10.1021/acs.jpclett.1c03437. 34791883

[ref63] Li Q.; Lian T. Ultrafast Charge Separation in Two-Dimensional CsPbBr3 Perovskite Nanoplatelets. J. Phys. Chem. Lett. 2019, 10 (3), 566–573. 10.1021/acs.jpclett.8b03610. 30642172

[ref64] Bekenstein Y.; Koscher B. A.; Eaton S. W.; Yang P.; Alivisatos A. P. Highly Luminescent Colloidal Nanoplates of Perovskite Cesium Lead Halide and Their Oriented Assemblies. J. Am. Chem. Soc. 2015, 137 (51), 16008–16011. 10.1021/jacs.5b11199. 26669631

[ref65] Do M.; Kim I.; Kolaczkowski M. A.; Kang J.; Kamat G. A.; Yuan Z.; Barchi N. S.; Wang L. W.; Liu Y.; Jurow M. J.; et al. Low-Dimensional Perovskite Nanoplatelet Synthesis using In Situ Photophysical Monitoring to Establish Controlled Growth. Nanoscale 2019, 11 (37), 17262–17269. 10.1039/C9NR04010B. 31246216

[ref66] Muljarov E. A.; Zhukov E. A.; Dneprovskii V. S.; Masumoto Y. Dielectrically Enhanced Excitons in Semiconductor-Insulator Quantum Wires: Theory and Experiment. Phys. Rev. B 2000, 62 (11), 7420–7432. 10.1103/PhysRevB.62.7420.

[ref67] Ghribi A.; Ben Aich R.; Boujdaria K.; Barisien T.; Legrand L.; Chamarro M.; Testelin C. Dielectric Confinement and Exciton Fine Structure in Lead Halide Perovskite Nanoplatelets. Nanomaterials 2021, 11 (11), 3054 10.3390/nano11113054. 34835818 PMC8621522

[ref68] Tao W.; Zhang C.; Zhou Q.; Zhao Y.; Zhu H. Momentarily Trapped Exciton Polaron in Two-Dimensional Lead Halide Pperovskites. Nat. Commun. 2021, 12 (1), 1400 10.1038/s41467-021-21721-3. 33658515 PMC7930248

[ref69] Marjit K.; Ghosh G.; Ghosh S.; Sain S.; Ghosh A.; Patra A. Structural Analysis and Carrier Relaxation Dynamics of 2D CsPbBr3 Nanoplatelets. J. Phys. Chem. C 2021, 125 (22), 12214–12223. 10.1021/acs.jpcc.1c03236.

[ref70] Yang W.; Yang Y.; Kaledin A. L.; He S.; Jin T.; McBride J. R.; Lian T. Surface Passivation Extends Single and Biexciton Lifetimes of InP Quantum Dots. Chem. Sci. 2020, 11 (22), 5779–5789. 10.1039/D0SC01039A. 32832054 PMC7416692

[ref71] Wu K.; Liang G.; Shang Q.; Ren Y.; Kong D.; Lian T. Ultrafast Interfacial Electron and Hole Transfer from CsPbBr3 Perovskite Quantum Dots. J. Am. Chem. Soc. 2015, 137 (40), 12792–12795. 10.1021/jacs.5b08520. 26414242

[ref72] Shang Q.; Piercy B. D.; Losego M. D.; Lian T. Effect of Surface Ligand on Charge Separation and Recombination at CsPbI3 Perovskite Quantum Dot/TiO2 Interfaces. J. Phys. Chem. C 2019, 123 (35), 21415–21421. 10.1021/acs.jpcc.9b06725.

[ref73] Villamil Franco C.; Mahler B.; Cornaggia C.; Gustavsson T.; Cassette E. Auger Recombination and Multiple Exciton Generation in Colloidal Two-Dimensional Perovskite Nanoplatelets: Implications for Light-Emitting Devices. ACS Appl. Nano Mater. 2021, 4 (1), 558–567. 10.1021/acsanm.0c02868.

[ref74] Makarov N. S.; Guo S.; Isaienko O.; Liu W.; Robel I.; Klimov V. I. Spectral and Dynamical Properties of Single Excitons, Biexcitons, and Trions in Cesium-Lead-Halide Perovskite Quantum Dots. Nano Lett. 2016, 16 (4), 2349–2362. 10.1021/acs.nanolett.5b05077. 26882294

[ref75] Li Q.; Yang Y.; Que W.; Lian T. Size- and Morphology-Dependent Auger Recombination in CsPbBr3 Perovskite Two-Dimensional Nanoplatelets and One-Dimensional Nanorods. Nano Lett. 2019, 19 (8), 5620–5627. 10.1021/acs.nanolett.9b02145. 31244208

[ref76] Socie E.; Vale B. R. C.; Burgos-Caminal A.; Moser J. E. Direct Observation of Shallow Trap States in Thermal Equilibrium with Band-Edge Excitons in Strongly Confined CsPbBr3 Perovskite Nanoplatelets. Adv. Opt Mater. 2021, 9 (1), 2001308 10.1002/adom.202001308.

[ref77] Mandal S.; George L.; Tkachenko N. V. Charge Transfer Dynamics in CsPbBr3 Perovskite Quantum Dots-Anthraquinone/Fullerene (C60) Hybrids. Nanoscale 2019, 11 (3), 862–869. 10.1039/C8NR08445A. 30600826

[ref78] Mandal S.; Tkachenko N. V. Multiphoton Excitation of CsPbBr3 Perovskite Quantum Dots (PQDs): How Many Electrons Can One PQD Donate to Multiple Molecular Acceptors?. J. Phys. Chem. Lett. 2019, 10 (11), 2775–2781. 10.1021/acs.jpclett.9b01045. 31071259 PMC6750835

[ref79] Li Q.; Lian T. Exciton Dissociation Dynamics and Light-Driven H2 Generation in Colloidal 2D Cadmium Chalcogenide Nanoplatelet Heterostructures. Nano Res. 2018, 11 (6), 3031–3049. 10.1007/s12274-018-2024-x.

[ref80] Huang J.; Huang Z.; Jin S.; Lian T. Exciton Dissociation in CdSe Quantum Dots by Hole Transfer to Phenothiazine. J. Phys. Chem. C 2008, 112 (49), 19734–19738. 10.1021/jp808291u.

[ref81] Alkaitis S. A.; Beck G.; Graetzel M. Laser Photoionization of Phenothiazine in Alcoholic and Aqueous Micellar Solution. Electron Transfer from Triplet States to Metal Ion Acceptors. J. Am. Chem. Soc. 1975, 97 (20), 5723–5729. 10.1021/ja00853a015.

[ref82] Shang Q.; Kaledin A. L.; Li Q.; Lian T. Size Dependent Charge Separation and Recombination in CsPbI3 Perovskite Quantum Dots. J. Chem. Phys. 2019, 151 (7), 074705 10.1063/1.5109894. 31438693

[ref83] Luo X.; Han Y.; Chen Z.; Li Y.; Liang G.; Liu X.; Ding T.; Nie C.; Wang M.; Castellano F. N.; et al. Mechanisms of Triplet Energy Transfer Across the Inorganic Nanocrystal/Organic Molecule Interface. Nat. Commun. 2020, 11 (1), 28 10.1038/s41467-019-13951-3. 31911606 PMC6946700

[ref84] Luo X.; Liang G.; Han Y.; Li Y.; Ding T.; He S.; Liu X.; Wu K. Triplet Energy Transfer from Perovskite Nanocrystals Mediated by Electron Transfer. J. Am. Chem. Soc. 2020, 142 (25), 11270–11278. 10.1021/jacs.0c04583. 32479073

[ref85] He S.; Li Q.; Jin T.; Lian T. Contributions of Exciton Fine Structure and Hole Trapping on the Hole State Filling Effect in the Transient Absorption Spectra of CdSe Quantum Dots. J. Chem. Phys. 2022, 156 (5), 054704 10.1063/5.0081192. 35135264

[ref86] Gramlich M.; Swift M. W.; Lampe C.; Lyons J. L.; Doblinger M.; Efros A. L.; Sercel P. C.; Urban A. S. Dark and Bright Excitons in Halide Perovskite Nanoplatelets. Adv. Sci. 2022, 9 (5), 2103013 10.1002/advs.202103013. PMC8844578 34939751

[ref87] Becker M. A.; Vaxenburg R.; Nedelcu G.; Sercel P. C.; Shabaev A.; Mehl M. J.; Michopoulos J. G.; Lambrakos S. G.; Bernstein N.; Lyons J. L.; et al. Bright Triplet Excitons in Caesium Lead Halide Perovskites. Nature 2018, 553 (7687), 189–193. 10.1038/nature25147. 29323292

[ref88] Sercel P. C.; Lyons J. L.; Wickramaratne D.; Vaxenburg R.; Bernstein N.; Efros A. L. Exciton Fine Structure in Perovskite Nanocrystals. Nano Lett. 2019, 19 (6), 4068–4077. 10.1021/acs.nanolett.9b01467. 31088061

[ref89] Yin J.; Li H.; Cortecchia D.; Soci C.; Brédas J.-L. Excitonic and Polaronic Properties of 2D Hybrid Organic–Inorganic Perovskites. ACS Energy Lett. 2017, 2 (2), 417–423. 10.1021/acsenergylett.6b00659.

[ref90] Cannelli O.; Colonna N.; Puppin M.; Rossi T. C.; Kinschel D.; Leroy L. M. D.; Loffler J.; Budarz J. M.; March A. M.; Doumy G.; et al. Quantifying Photoinduced Polaronic Distortions in Inorganic Lead Halide Perovskite Nanocrystals. J. Am. Chem. Soc. 2021, 143 (24), 9048–9059. 10.1021/jacs.1c02403. 34075753 PMC8227469

[ref91] Guzelturk B.; Winkler T.; Van de Goor T. W. J.; Smith M. D.; Bourelle S. A.; Feldmann S.; Trigo M.; Teitelbaum S. W.; Steinruck H. G.; de la Pena G. A.; et al. Visualization of Dynamic Polaronic Strain Fields in Hybrid Lead Halide Perovskites. Nat. Mater. 2021, 20 (5), 618–623. 10.1038/s41563-020-00865-5. 33398119

[ref92] Kobosko S. M.; DuBose J. T.; Kamat P. V. Perovskite Photocatalysis. Methyl Viologen Induces Unusually Long-Lived Charge Carrier Separation in CsPbBr3 Nanocrystals. ACS Energy Lett. 2020, 5 (1), 221–223. 10.1021/acsenergylett.9b02573.

[ref93] Luo X.; Liang G.; Wang J.; Liu X.; Wu K. Picosecond Multi-Hole Transfer and Microsecond Charge-Separated States at the Perovskite Nanocrystal/Tetracene Interface. Chem. Sci. 2019, 10 (8), 2459–2464. 10.1039/C8SC04408B. 30881674 PMC6385846

[ref94] Zhao C.; Tian W.; Sun Q.; Yin Z.; Leng J.; Wang S.; Liu J.; Wu K.; Jin S. Trap-Enabled Long-Distance Carrier Transport in Perovskite Quantum Wells. J. Am. Chem. Soc. 2020, 142 (35), 15091–15097. 10.1021/jacs.0c06572. 32786774

[ref95] Zhu H.; Song N.; Rodriguez-Cordoba W.; Lian T. Wave Function Engineering for Efficient Extraction of up to Nineteen Electrons from One CdSe/CdS Quasi-Type II Quantum Dot. J. Am. Chem. Soc. 2012, 134 (9), 4250–4257. 10.1021/ja210312s. 22329340

