# Supplementary material for: Electron Trapping Prolongs the Lifetime of Charge-Separated States in 2D Perovskite Nanoplatelet-Hole Acceptor Complexes
Source: J Phys Chem Lett. 2023 Feb 23;14(9):2241–50. doi: 10.1021/acs.jpclett.2c03815 (PMC10009813; doi:10.1021/acs.jpclett.2c03815)
Supplement: Supplementary file 1 — jz2c03815_si_001.pdf [file jz2c03815_si_001.pdf]

Supporting Information for

Electron Trapping Prolongs the Lifetime of Charge Separated States in 2D Perovskite Nanoplatelet-Hole Acceptor Complexes

Sheng He, Tao Jin, Anji Ni, Tianquan Lian*

Department of Chemistry, Emory University, 1515 Dickey Drive Northeast, Atlanta, Georgia 30322, USA

Content List:

S1. Sample preparation

S2. Sample characterization

S3. Dielectric confinement change induced by surface passivation

S4. PL lifetime measurement and data analysis

S5. Transient absorption (TA) setup

S6. TA spectra of NPLE

S7. Power dependence of the XB decay kinetics

S8. Energy level alignment between NPL and charge acceptors

S9. Electron transfer from NPLA to MV^{2+} and AQA

S10. Calculation of the average number of PTZ on each NPLA and NPLE

S11. PL quench by hole transfer to PTZ

S12. Supporting TA results for hole transfer

S13. Fitting of the XB decay kinetics of NPLA and NPLE

S14. Estimation of the electron and hole contribution to the XB signal

S15. Kinetics fitting of NPLA-PTZ and NPLE-PTZ

S16. The electron trapping-detrappping model

S17. Estimation of the detrapping lifetime and trap depth

S1. Sample preparation

Materials. Lead bromide ($PbBr_2$, 99.999 %, Sigma-Aldrich), cesium carbonate (Cs_2CO_3, Sigma-Aldrich), oleic acid (OAc, 90 %, Sigma-Aldrich), oleylamine (70 %, Sigma-Aldrich), phenothiazine (PTZ, ≥ 98 %, Sigma-Aldrich), methyl viologen dichloride hydrate (MV, 98 %, Sigma-Aldrich), anthraquinone-2-carboxylic acid (AQA, 98 %, Sigma-Aldrich), toluene (Macron Fine Chemicals), hexane (98.5 %, Fisher Chemicals), 1-octadecene (ODE, 90 %, Sigma-Aldrich), Acetone (Fisher Chemicals).

Synthesis of NPLs was modified from literature.[1] Typically, the Cs-oleate and $PbBr_2$ precursors are first prepared. 0.1 mmol Cs_2CO_3 was added into 10 mL oleic acid. The mixture was stirred at 100 ̊C under Argon (Ar) atmosphere for around 30 minutes to dissolve the solid. The solution was then kept in air as the Cs-oleate precursor. The $PbBr_2$ precursor was prepared by dissolving 0.2 mmol $PbBr_2$, 200 μL oleic acid and 200 μL oleylamine in 20 mL toluene at 100 ̊C under Ar atmosphere. To synthesize the 3 monolayer (ML) NPLs, 1.5 mL of the $PbBr_2$ precursor was loaded into a 20 mL vial and stirred at 1200 rpm. 150 μL of the Cs-oleate precursor was added in to the $PbBr_2$ precursor and stirred for 10 seconds. 2 mL Acetone was then quickly injected into the precursor mixture to induce the formation of NPLs. The mixture was stirred for another 1 minutes to allow for the complete reaction. The resulting NPLs was then separated from the reactants by centrifuging the mixture at 4000 rpm for 3 minutes. The precipitated NPLs were finally redispersed in 2 mL Hexane for future characterization. For 2 ML NPLs, 3 mL $PbBr_2$ precursor was used while others steps were the same.

Surface passivation of NPLs followed reported method with minor modifications.[1-2] The $PbBr_2$ precursor was used as the passivation (enhancement) solution. Typically, 0.1 mL $PbBr_2$ precursor was added into 0.9 mL hexane solution of NPLs. The mixture was shaken for 5 seconds to uniformly distribute the $PbBr_2$ on the NPL surface. Bright blue emission was observed immediately after passivation. For comparison between the pristine and passivated NPLs in future spectroscopy experiments, pristine NPLs samples are also dispersed in the 9: 1 hexane: toluene solution.

Preparation of NPLA(E)-Acceptor complexes. Excessive powder of charge acceptor molecules (PTZ, MV, AQA) were added into the hexane solution of the NPLs, followed by sonication of 20 minutes. The resulted mixture was then filtered through a polyethersulfone syringe filter with a pore size of 200 nm to remove undissolved molecules. Since MV and AQA are not soluble in hexane, any remaining molecules in the solution are believed to adsorb on the NPL surface. The loading of PTZ on the NPL surface is discussed in section S10.

S2. Sample characterization

Spectroscopy measurements of the NPL samples were taken under ambient condition in quartz cuvettes with a light path of 1 mm. The UV-Vis absorption spectra were taken using the Agilent 8453 spectrometer, and the photoluminescence spectra were taken using the Horiba Scientific Fluoromax-plus spectrometer.

Morphology and size of the samples in the main text are characterized by Hitachi HT-7700 transmission electron microscope (TEM) operating at 80 kV. The TEM images are shown in Figure S1, and the size distribution histograms are given in Figure S2. Note that the measured thickness of the NPLs (2.9 ± 0.4 nm, from the NPLA-PTZ sample, shown in Figure S1b, S1c, and S2c) is inconsistent with the theoretical value of the 3 ML NPLs (1.8 nm for 3 layers of lead bromide octahedra)[2] due to possible non-perpendicular standing of the NPL stacks on the substrate, resulting in an overestimation of the NPL thickness.[3] Nonetheless, the number of monolayers in the NPLs can be inferred from the position of the band edge exciton transition peak in ground state UV-Vis absorption spectra, as discussed in the main text. The slightly different lengths measured in Figure S1a and S1d suggest possible NPL volume growth after passivation, consistent with the absorption spectrum of NPLE discussed in Figure 1c.

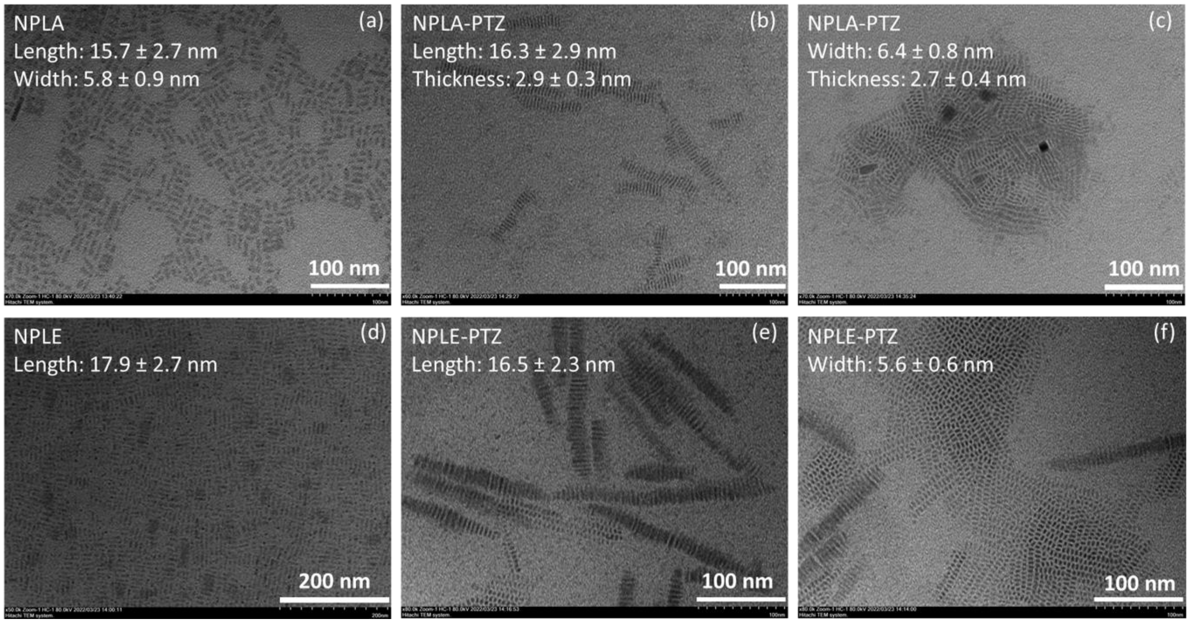

Figure S1. TEM images of the NPL samples. (a) NPLAs laying down on the substrate. (b) and (c) NPLA-PTZ sample in the self-assembled face-to-face stack and standing on the long and short edge of the rectangular shape, respectively. (d) Self-assembled NPLE sample standing on the long edge. (e) and (f) Self-assembled NPLE-PTZ sample standing on the long and short edge, respectively.

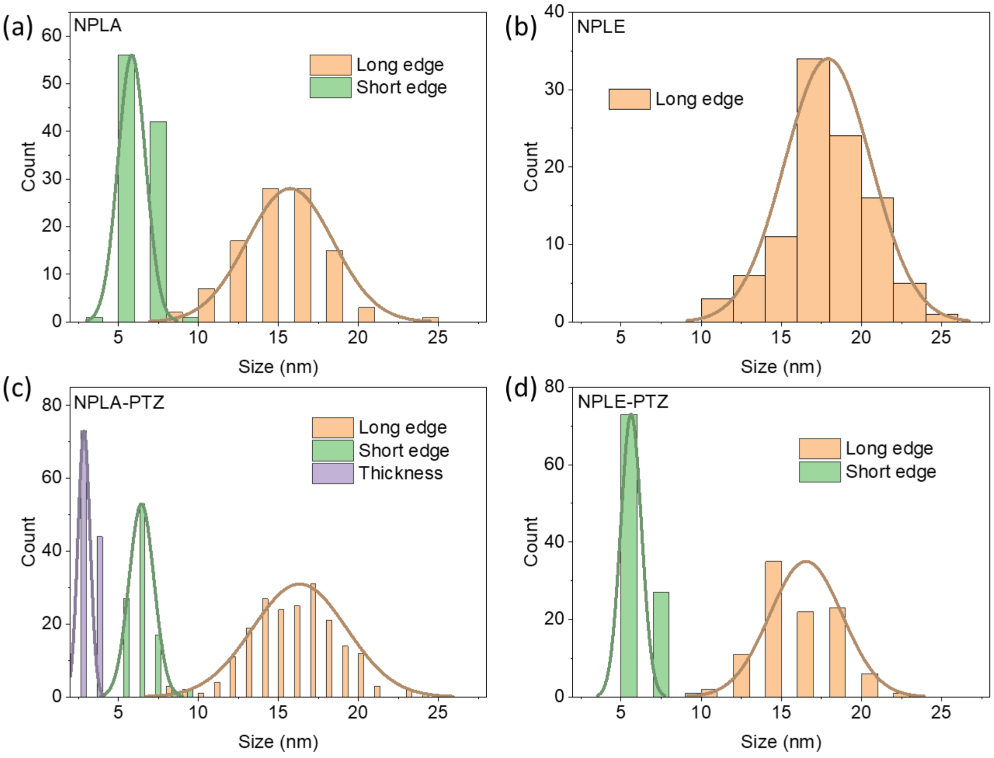

Figure S2. Size distribution of (a) NPLA, (b) NPLE, (c) NPLA-PTZ, and (d) NPLE-PTZ. The passivation or the addition of PTZ causes negligible changes to the size and morphology of the NPLs.

S3. Dielectric confinement change induced by surface passivation

As shown in Figure S3a, diluting the NPLE sample with pure hexane (denoted by NPLEH) causes minor red shift of the exciton transition (from 443 nm to 446 nm) and the continuous absorption band. On the other hand, Figure S3b shows that diluting the NPLE sample with a ligand solution (4 mM oleic acid and oleylamine in hexane, denoted by NPLEL) results in significant blue shift of the transitions. Note that the NPL thickness is believed to be maintained during the dilution, since the spectrum shape is not changed. If the blue shift is induced by thickness decrease, or the formation of 2 ML NPLs, the relative height of the continuous absorption band would decrease significantly, as shown by the spectrum of 2 ML NPLs (black dashed line in Figure S3b).[2, 4] These results indicate that more ligands on the NPL surface will increase the energy of inter-band transitions by increasing the dielectric confinement of the NPLs.[5]

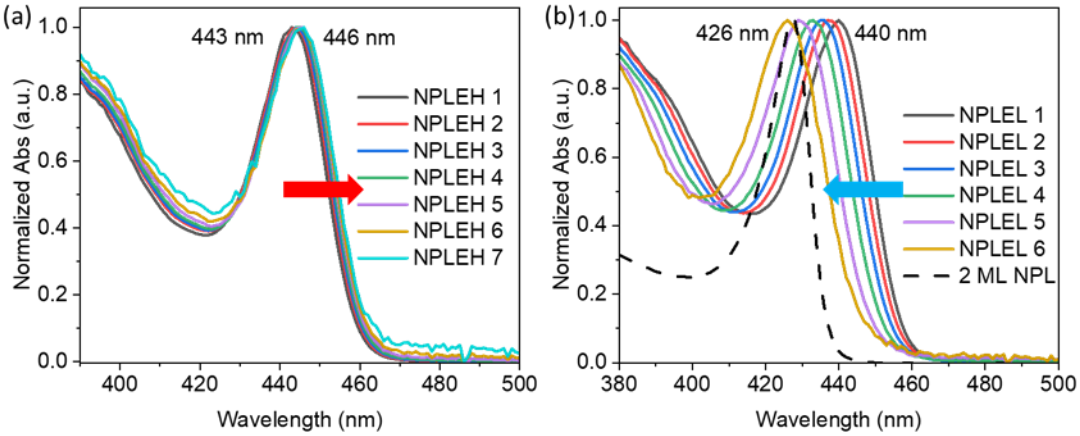

Figure S3. Absorption spectra of NPLE samples diluted in (a) pure hexane and (b) ligand solution. The absorption energy of the NPLs shows red (blue) shift with increasing dilution in hexane (ligand solution). The spectrum of as-synthesized 2 ML NPL is shown in (b) as the black dashed line.

S4. PL lifetime measurement and data analysis

Time-resolved photoluminescence (TRPL) setup is built with a mode-locked Ti: Sapphire laser (Tsunami oscillator, Spectra Physics). The laser output is selected as 800 nm with a repetition rate of 80 MHz. The repetition rate is reduced to 6.67 MHz by an E-O modulator (ConOptics, Model 350-160), resulting in a 150 ns time scale for PL lifetime measurements. The laser pulse is then directed into a BBO crystal to generate the 400 nm laser through the second harmonic generation effect. The 400 nm laser is then used as the excitation pulse to generate the PL. The emitted PL photons from the sample are filtered through a 425 nm long-pass dichroic mirror and then collected into a monochromator (Acton Series, Princeton Instruments), which can selectively pass PL photons at the PL spectra peak wavelength. After the monochromator, the selected photons were collected by a microchannel-plate-photomultiplier tube (Hamamatsu R3809U-51), which is connected to a time-correlated single photon counting (TCSPC) board (Becker & Hickel SPC 600) to generate the PL decay data.

The PL decay curves are normalized by the number of absorbed photons to compare the relative PL intensity I_{PL} between different samples, as given by Equation S1.

$$I_{PL} = c \cdot \frac{\int_0^{+\infty} I(t)dt}{t_{collect} \cdot A} \qquad (S1)$$

In Equation S1, $I(t)$ is the PL intensity at delay time t after the excitation, $t_{collect}$ is the collection time of the PL lifetime measurement, A is the absorptance of the sample at 400 nm, and c is the instrument parameter, including the collection efficiency of the detector and the photon flux of the excitation. Comparing the I_{PL} of NPLA and NPLE generates a PL enhancement factor of 3.25, consistent with the 2 ~ 7 enhancement factor in literature using similar passivation method.[2]

The PL decay curves of NPLA and NPLE are fitted to three and two exponential decay functions, respectively, convoluted with the instrument response function (IRF), as given in Equation S2, where A_i is the amplitude of ith exponential decay component, τ_i is the decay lifetime constant, and I_0 is a constant

offset caused by noise in the measurement. The amplitude weighted average lifetime is calculated by Equation S3. The fitting results are summarized in Table S1.

$$I(t) = IRF \otimes \left(\sum_{i=1}^{m} A_i e^{-\frac{t}{\tau_i}} + I_0 \right) \qquad (S2)$$

$$\bar{\tau} = \frac{\sum_i A_i \tau_i}{\sum_i A_i} \qquad (S3)$$

Table S1. Fitting parameters of the PL decay curves.

	NPLA		NPLE	
i	A_i/a.u.	τ_i/ns	A_i/a.u.	τ_i/ns
1	21.7 ± 0.2	0.28 ± 0.01	15.3 ± 1.0	2.73 ± 0.10
2	18.1 ± 0.2	1.72 ± 0.04	19.1 ± 1.0	6.24 ± 0.12
3	1.8 ± 0.3	5.67 ± 0.43		
Average		1.14 ± 0.04		4.68 ± 0.11

PL quantum yields (PLQY) of NPLE is measured by comparing the PL intensity with that of the standard organic dye molecule Coumarin 343 (C343) under 400 nm excitation. The PLQY of C343 dissolved in ethanol is 63 %.[6] The PLQY of NPLE is calculated to be 32.3 ± 0.7 %. PLQY of NPLA is then calculated by the above PL enhancement factor.

S5. Transient absorption (TA) setup

Femtosecond TA setup. In the femtosecond TA experiments, we used a regenerative amplified Ti: Sapphire femtosecond laser system (Astrella, Coherent) to generate the 800 nm fundamental pulse (1 kHz repetition rate, 35 fs pulse duration, and 5.1 mJ/pulse), which was split by a 90:10 beam splitter. The 10% 800 nm fundamental pulse went through a delay stage (with a maximum delay time of 8 ns) in the Helios system (Ultrafast System LLC) and was then attenuated by a neutral-density filter before it was focused on a sapphire to generate a white light continuum (WLC, from 420 nm to 780 nm) probe pulse. The WLC was focused on the sample to monitor the absorbance change. For the 90 % fundamental beam, half of it was used to pump a visible optical parameter amplifier (OPA, Light Conversion LLC) to generate the pump pulse with desired wavelength in the visible range. For the 400 nm pump pulse, the 800 nm fundamental pulse was frequency-doubled by a BBO crystal. The output pump pulse was then split by a 50:50 beam splitter. The reflected half was guided into the nanosecond TA setup, as discussed below. The transmitted half was directed into the Helios system and then modulated by a 500 Hz chopper before it

was focused on the sample. The power of the pump pulse was controlled by a series of neutral-density filters. The data collection was completed in the Helios system (Ultrafast System LLC), with an instrument response function (IRF) of 70 fs. All samples were contained in 1 mm thick quartz cuvettes and were constantly stirred. All measurements were conducted at room temperature.

Nanosecond TA setup. Nanosecond TA was performed with the EOS spectrometer (Ultrafast Systems LLC). The pump beam was generated by the same OPA used in the femtosecond TA setup. A supercontinuum laser (Disco-2-UV, Leukos) was use as the WLC probe. The WLC was split to a probe beam and a reference beam, which were then focused into a fiber-coupled multichannel spectrometer with complementary metal-oxide-semiconductor (CMOS) sensors. The function of this reference beam was to account for the pulse-to-pulse fluctuation of the WLC. The delay time between the pump and probe beam was controlled by a digital delay generator (CNT-90, Pendulum Instruments). The IRF of this system was measured to be 400 ps, giving an overlapped delay time window from 1 ns to 7 ns between the femtosecond and nanosecond TA measurements. Since both the femtosecond and the nanosecond TA data at long delay time (> 1 ns) represent the single exciton state,[7] the kinetics data from these two TA measurements were connected by scaling the nanosecond data to match the femtosecond data within the overlapped delay time window.

S6. TA spectra of NPLE

The TA spectra of NPLE were measured under the same condition as that of NPLA. The spectra are shown in Figure S4, where the XB signal centers at 446 nm. Compared to NPLA, the TA features in NPLE blue shifted, consistent with the ground state absorption result in Figure 1c.

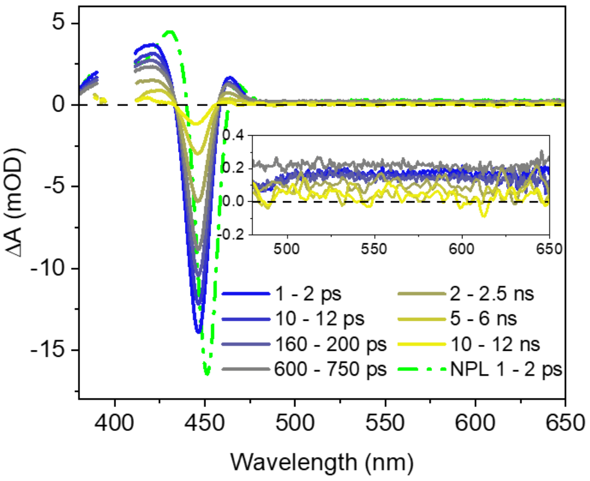

Figure S4. TA spectra of NPLE at indicated delay times following excitation. The TA spectrum of NPLA averaged from 1ps to 2 ps is shown as green dash dot line for direct comparison. The inset shows a zoom-in view from 480 nm to 650 nm.

S7. Power dependence of the XB decay kinetics

To study the fast decay of the XB in both NPLA and NPLE, the power of the 400 nm excitation was lowered. As shown in Figure S5, the XB kinetics remain the same even when the XB amplitude was reduced by 66% (inset in Figure S5), showing no power dependence under the experimental conditions. Thus, the fast decay in 20 ps was attributed to intrinsic properties of the NPL rather than the Auger type recombination.

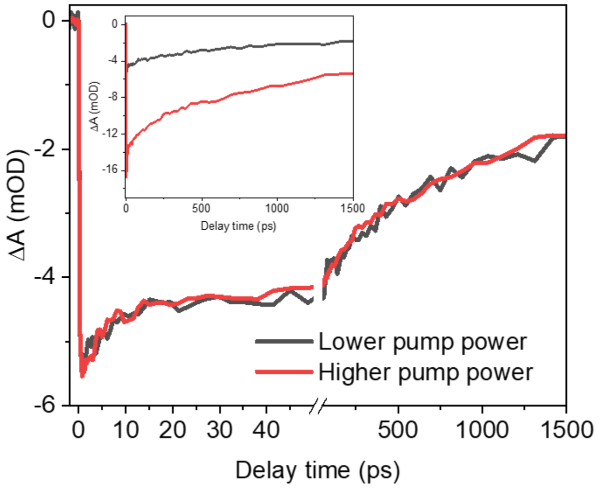

Figure S5. Scaled XB decay kinetics under different pump power. The inset shows XB amplitude difference before scaling.

S8. Energy level alignment between NPL and charge acceptors

The band edge positions in the 3 ML NPL in Figure 3a were estimated following our previous publication[8] with updated material parameters. The bandgap and the VB top of bulk $CsPbBr_3$ was taken as 2.36 eV[9] and -5.60 eV[10] (relative to vacuum), respectively, and the CB bottom in bulk was calculated to be -3.24 eV. The CB bottom (VB top) in the NPL was then calculated to be -3.04 eV (-5.80 eV) due to the strong confinement in thickness.[8] Figure 3a compares the band edges of NPL with the highest occupied molecular orbital (HOMO) of PTZ (-5.50 eV)[11] and the lowest unoccupied molecular orbital (LUMO) of MV (-4.0 eV)[12] and AQA (-3.46 eV)[13] to show the energetically allowed charge transfer steps. In addition, the triplet state energies (E_T) of these charge acceptors are listed here: 2.62 eV in PTZ, 3.0 eV in MV, and 2.7 eV in AQA.[14] These acceptors were selected with E_T comparable or larger than the exciton energy of the NPL (2.76 eV) to avoid triplet energy transfer from the NPL, which may complicate the charge separation and recombination kinetics.[15-16] Note that although the E_T of PTZ is smaller than the exciton energy, our spectral analysis showed no triplet PTZ formation after hole transfer, as discussed in Figure S11, section S12.

S9. Electron transfer from NPLA to MV^{2+} and AQA

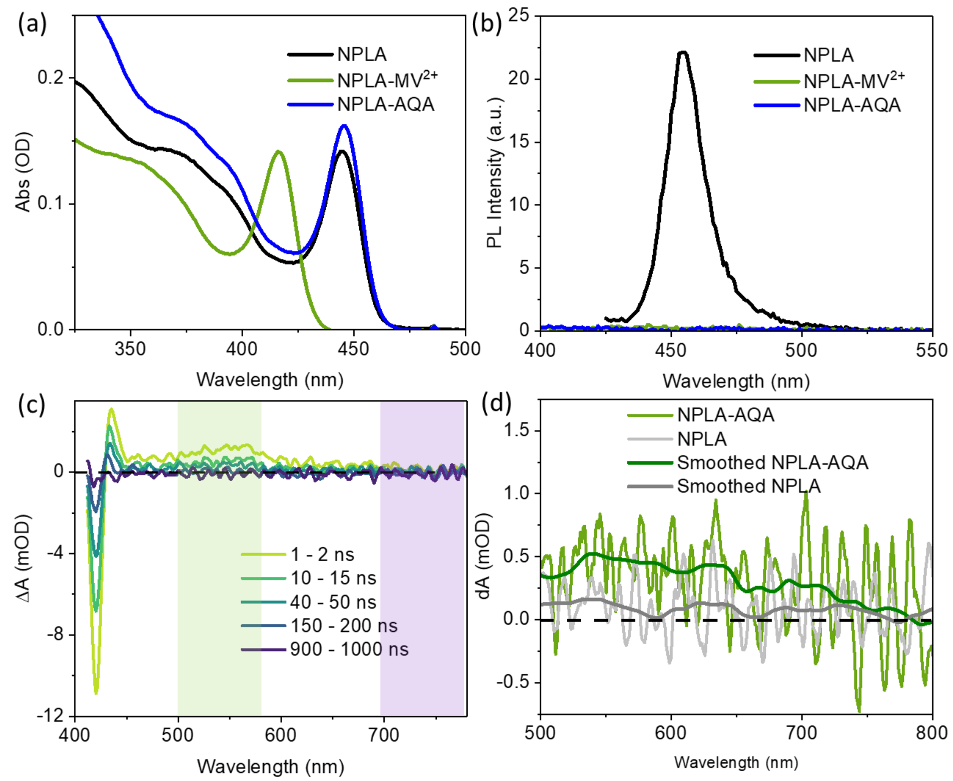

Figure S6. Absorption (a) and PL (b) spectra of NPLA-MV^{2+} and NPLA-AQA. The PL spectra were collected by exciting the NPLA-MV^{2+} and NPLA-AQA at 350 nm and exciting NPLA at 400 nm. (c) TA spectra of NPLA-MV^{2+} under 400 nm excitation. The $MV^{+\bullet}$ signal is indicated by the light green shadow. (d) Original spectra of the PA signal at 1.5~2 ns in NPLA-AQA and NPLA. The smoothed data in Figure 3b inset is also shown.

The absorption and PL spectra of NPLA-MV^{2+} and NPLA-AQA are shown in Figure S6a and S6b, respectively, together with the spectra of pure NPLA. Adsorption of MV^{2+} and AQA caused negligible changes to the absorption spectral shape of NPLAs. The blue shift in NPLA- MV^{2+} was attributed to the anion exchange between the Cl^- in MV and Br^- in NPLA.[17] The PL spectra shows near unity PL quenching in NPLA- MV^{2+} and NPLA-AQA, suggesting efficient ET from NPLA to MV^{2+} and AQA.[13, 18] TA spectra of NPLA- MV^{2+} and NPLA-AQA in the nanosecond delay window are shown in Figure S6c and Figure 3b, respectively, both of which show broad positive peaks to the red of the XB of NPLA (500 ~ 550 nm in NPLA- MV^{2+}, 550 ~ 650 nm in NPLA-AQA). These positive peaks in TA spectra are characteristic of reduced MV^{2+} ($MV^{+\bullet}$)[18-19] and AQA (AQA^-).[13] Kinetics of the XB and the acceptors are compared in Figure 3c and d. The AQA^- kinetics in Figure 3c was obtained by subtracting the averaged kinetics between 750 nm and 850 nm from that between 550 nm and 650 nm. The $MV^{+\bullet}$ kinetics was obtained by subtracting the averaged kinetics between 700 nm and 780 nm (light purple shadow in Figure S6c) from that averaged between 500 nm and 580 nm (light green shadow in Figure S6c). In both Figure

3c and d, the acceptors' kinetics are flipped and scaled to compare with the XB kinetics after 100 ps. The acceptors' kinetics in the picosecond window are not shown due to large noise. In both NPLA- MV^{2+} and NPLA-AQA, the XB kinetics show an ultrafast (< 5 ps) decay corresponding to the ET and then a long-lived amplitude contributed by the remaining hole in the NPLA, which decay with the same rate as the reduced acceptors. The long-lived hole-induced XB after ET indicates that there is almost no other decay pathways for the VB hole except decaying with the electron transferred to the acceptors.

The XB kinetics in NPLA- MV^{2+} and NPLA-AQA was fitted by Equation S4:

$$A_{XB,NPL-MV^{2+}/AQA}(t) = A_0\left(c_e e^{-\frac{t}{\tau_{ET}}} + c_{h1} e^{-\frac{t}{\tau_{CR1}}} + c_{h2} e^{-\frac{t}{\tau_{CR2}}}\right)\otimes IRF \qquad (S4)$$

In Equation S44, c_e, c_{h1} and c_{h2} are the electron and hole contributions to the XB, τ_{ET} is the ET lifetime, τ_{CR1} and τ_{CR2} are the charge recombination lifetimes. Two-exponential decay was found necessary to fit the charge recombination part, possibly due to the heterogeneous distribution of acceptors on the NPL surface. The fitting results are represented by the red lines in Figure 3c and d. The fitting parameters and the amplitude-weighted average charge recombination lifetime τ_{avg} are listed in Table S2. The fitted c_e is slightly less than 50% (see section S14), which may be caused by the initial amplitude loss due to the convolution of the IRF with the ultrafast ET. Both ET lifetime in NPLA- MV^{2+} (4 ps) and NPLA-AQA (0.5 ps) are shorter than the electron trapping lifetimes (9 ps and 516 ps, section S13), indicating that electron traps may not affect the ET or exciton dissociation.

Table S2. Fitting parameters of the NPLA- MV^{2+} and NPLA-AQA XB kinetics

	NPLA- MV^{2+}	NPLA-AQA
A_0/mOD	-3.38 ± 0.05	-5.66 ± 0.16
c_e	44.6 ± 0.9 %	48.9 ± 1.4 %
τ_{ET}/ps	4.05 ± 0.31	0.49 ± 0.04
c_{h1}	14.8 ± 1.3 %	29.6 ± 1.7 %
τ_{CR1}/ns	3.8 ± 0.8	4.0 ± 0.3
c_{h2}	40.6 ± 1.3 %	21.5 ± 1.7 %
τ_{CR2}/ns	96.2 ± 5.8	38.2 ± 3.5
τ_{avg}/ns	71.6 ± 4.5	18.4 ± 1.6

S10. Calculation of the average number of PTZ on each NPLA and NPLE

12 mg of PTZ powder was added into the solution of NPLA or NPLE, which was then sonicated for 20 minutes, followed by filtration to remove undissolved PTZ solid. TEM characterization of NPLA-PTZ and NPLE-PTZ (Figure S1 and S2) shows no shape or size change of the NPLA or NPLE after PTZ adsorption. Figure S8a shows the absorption spectrum of NPLE-PTZ. To obtain the absorption of PTZ in NPLE-PTZ, the absorption spectrum of NPLE was first scaled to the same exciton peak intensity in NPLE-PTZ. Then the scaled spectrum of NPLE was subtracted from the spectrum of NPLE-PTZ. The PTZ absorption in NPLA-PTZ in Figure 4a was obtained in the same way.

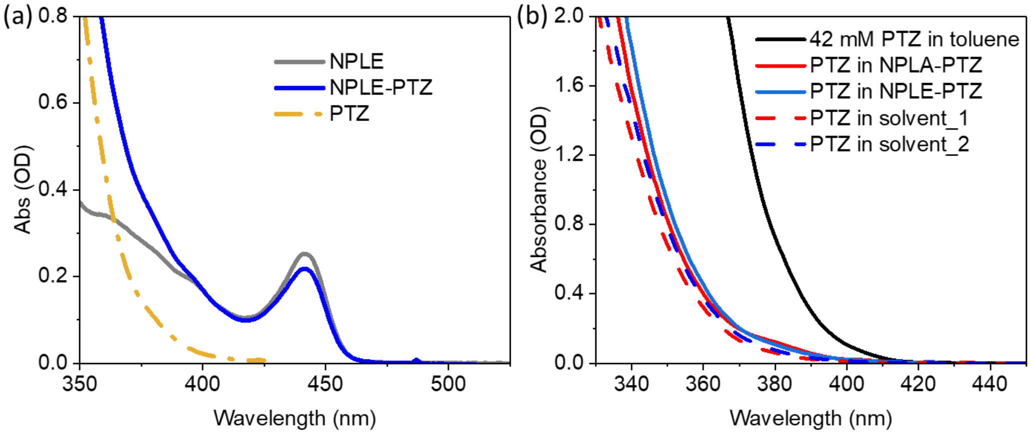

Figure S7. (a) Absorption spectra of NPLE-PTZ and the PTZ component, together with the spectrum of pure NPLE. (b) PTZ absorption in standard solution, NPLA-PTZ, NPLE-PTZ, the solvent of NPLA-PTZ without NPLA (solvent_1), and the solvent of NPLE-PTZ without NPLE (solvent_2). All spectra were measured in 1 mm thick cuvette.

Since the NPLA is dispersed in 9: 1 hexane: toluene mixture solvent (section S1), part of the PTZ molecules are dissolved in toluene while the other part on NPLA surface. To subtract the PTZ in toluene, the same procedure of loading PTZ was repeated in a pure 9: 1 hexane: toluene solvent without NPLA (solvent_1). Similar experiment was repeated for NPLE-PTZ using the 9:1 hexane: $PbBr_2$ precursor solvent without NPLE (solvent_2).

The absorption spectra of PTZ in NPLA-PTZ, NPLE-PTZ, solvent_1, solvent_2, and a standard solution (42 mM PTZ in toluene) are shown in Figure S7b. The higher absorption of PTZ in NPLA-PTZ and NPLE-PTZ than that in mixed solvent indicates extra PTZ on NPLA and NPLE surface. According to the Beer-Lambert law, the concentration of PTZ on NPLA (NPLE) is 0.69 (0.74) mM. Note that the PTZ concentration in solvent_2 is higher than that in solvent_1, probably due to the extra organic ligand (oleic acid and oleylamine) in the $PbBr_2$ precursor solution, which increases PTZ's solubility in toluene.

The extinction coefficient of the NPL is estimated to be 0.22 $cm^{-1}\mu M^{-1}$ at 400 nm according to literature,[20] and the concentration of NPLA (NPLE) is 8.1 μM (7.8 μM) accordingly. The number of PTZ per NPL is 85 for NPLA and 95 for NPLE.

Alternatively, the extinction coefficient at the first excitonic peak of the 3 ML NPLs is measured to be 3.81 $cm^{-1}\mu M^{-1}$ via the combination of absorption spectrum, TEM, and the inductively coupled plasma mass spectrometry (ICP-MS). Specifically, 16.27 μg Pb is measured out of 0.2 mL new NPLA colloidal solution, the absorption of which is 0.384 at the first excitonic transition in a 2 mm thick cuvette. 780 $[PbBr_3]^-$ octahedron units in one NPLA are estimated from the TEM image (Figure S1a) by assuming that the length of one unit is 0.6 nm.[21] Then the concentration of NPLA and the extinction coefficient are calculated. The resulting NPL concentration is 0.614 μM for NPLA and 0.661 μM for NPLE in the main text. The number of PTZ per NPL is 1124 for NPLA and 1119 for NPLE.

The numbers of PTZ per NPL calculated from both methods are much larger than our previous report (< 4),[8] possibly due to the different sample preparation method or the overestimation of the NPL's extinction coefficient.[20, 22] Nevertheless, the numbers of PTZ on both NPLA and NPLE are similar, enabling a direct comparison of the HT kinetics in NPLA-PTZ and NPLE-PTZ.

S11. PL quench by hole transfer to PTZ

As discussed in section S4, the relative PL intensity can be represented by the integration of the PL decay curves. Figure S8 shows the PL decay curves of NPLA-PTZ and NPLE-PTZ, normalized by the number of absorbed photons. Comparing the integration with and without PTZ gives the PL quench efficiency of 93% and 97% in NPLA-PTZ and NPLE-PTZ, respectively.

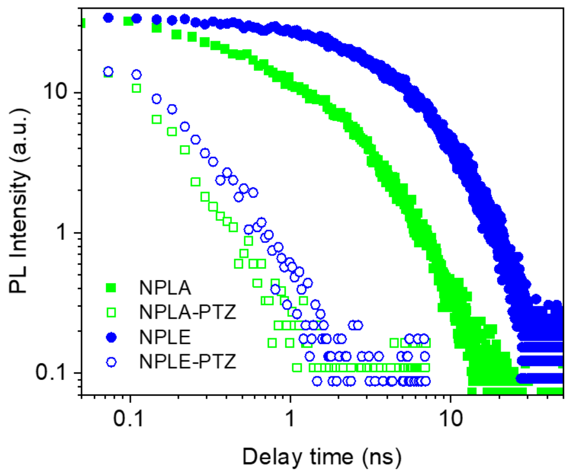

Figure S8. PL decay curves of NPLA, NPLA-PTZ, NPLE, and NPLE-PTZ. The PL intensities are normalized by the number of absorbed photons in each sample, as discussed in section S4.

S12. Supporting TA results for hole transfer

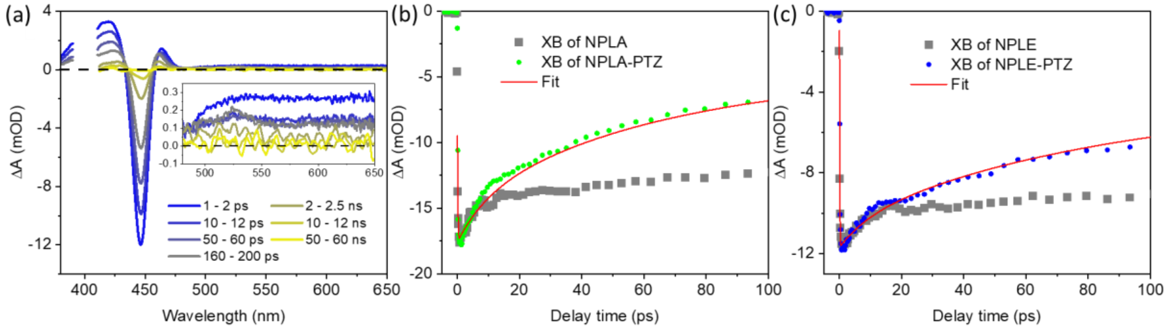

Figure S9. (a) TA spectra of NPLE-PTZ at indicated delay times following 400 nm excitation. The inset shows a zoom-in view of the spectra from 480 nm to 650 nm. A positive peak appears around 525 nm starting from 10 ps, corresponding to the PTZ^+ formation after hole transfer (HT). The related kinetics are shown in Figure 4d. (b) and (c) TA kinetics in Figure 4c and d in the range from -5 ps to 100 ps, showing the same decay kinetics of XB in pure NPL and NPL-PTZ within 10 ps caused by fast electron trapping.

The kinetics in NPLA-PTZ and NPLE-PTZ were confirmed using a new batch of NPL-PTZ complexes, denoted by NPLA-PTZ_2 and NPLE-PTZ_2. As shown in Figure S10a, after HT, the XB in NPLA-PTZ_2 decays faster than PTZ^+ from 0.5 ns to 20 ns, while a zoom-in view (inset in Figure S10a) shows the same decay kinetics of the PTZ^+ and the small, long tail of the XB after 20 ns, the same phenomenon observed in Figure 4c and Figure S13a for NPLA-PTZ. Figure S10b shows the XB and PTZ^+ kinetics in NPLE-PTZ_2. After HT (> 500 ps), the XB decays in the same rate as PTZ^+, consistent with electron transferring from the CB in NPLE_2 to PTZ^+, which is the same as in NPLE-PTZ (Figure 4d).

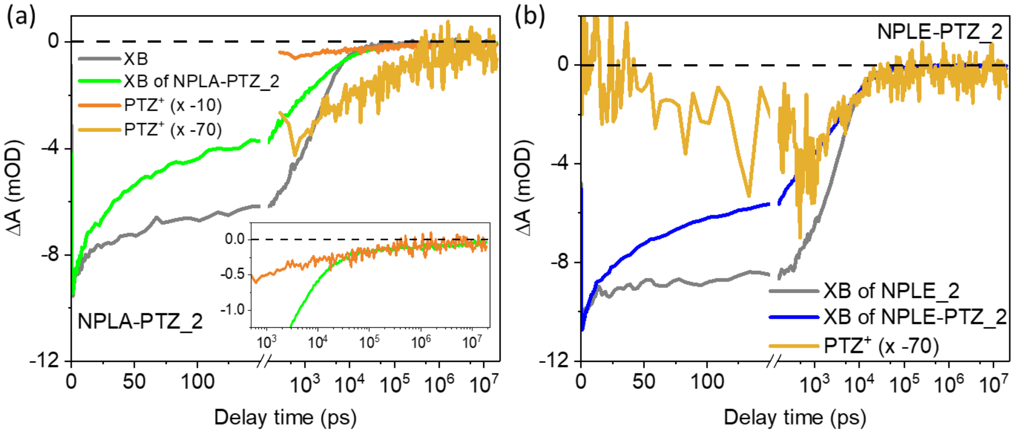

Figure S10. Confirmation of the kinetics in NPLA-PTZ and NPLE-PTZ. (a) XB kinetics of NPLA_2 and NPLA-PTZ_2, compared to the PTZ^+ kinetics in NPLA-PTZ_2 with large (x -70) and small (x -10) scaling. The PTZ^+ kinetics before 500 ps are not shown due to high noise level in the measurement. The inset shows a zoom-in view of the kinetics after 500 ps. (b) XB kinetics of NPLE_2 and NPLE-PTZ_2, compared to the scaled (x -70) PTZ^+ kinetics in NPLE-PTZ_2.

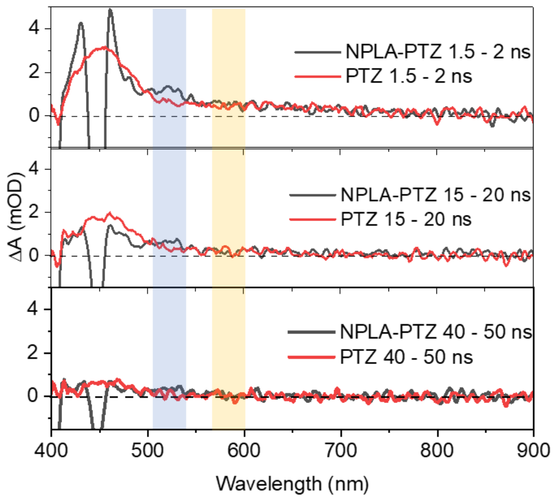

Figure S11. Comparison of the TA spectra of NPLA-PTZ and pure PTZ in toluene at different delay times after 400 nm excitation. The kinetics of PTZ^+ was taken as the difference between the averaged kinetics in the blue shadow (from 510 nm to 540nm) and the orange shadow (from 570 nm to 600 nm), minimizing the interference from the background. The same method was applied to the NPLE-PTZ data.

One possible reason for the discrepancy between the decay kinetics of XB and PTZ^+ in NPLA-PTZ may be the sequential charge transfer to form triplet in the acceptor.[15-16] For instance, Luo et al. reported that after electron transfer from $CsPbCl_3$ NCs to rhodamine B (RhB), the hole in the NC subsequently transfers to the reduced RhB to form the RhB triplet, causing the fast XB decay and the slow RhB ground state bleach decay.[15] However, in this study, the long-lived PTZ^+ PA signal in NPLA-PTZ excluded the formation of PTZ triplet by sequential electron transfer.[23] Control experiment also confirmed no PTZ triplet signal in the TA spectra of NPLA-PTZ (Figure S11). Figure S11 shows the TA spectra (red) of pure PTZ in toluene under 400 nm excitation. The positive peak around 460 nm is attributed to the absorption of PTZ triplet ($^3PTZ^*$) generated by intersystem crossing of PTZ singlet.[23] The comparison between the TA spectra of $^3PTZ^*$ and NPLA-PTZ (black), where most of the 400 nm photons were absorbed by NPLA, shows that there is negligible $^3PTZ^*$ in NPL-PTZ, as evidenced by the spectral shape difference from 450 nm to 500 nm. Instead, the spectra of NPLA-PTZ shows a positive peak from 510 nm to 540 nm, which is the absorption of PTZ^+ and absent in the spectra of pure PTZ. Furthermore, in the case of PTZ^+ decay and $^3PTZ^*$ formation, a positive feature around 460 nm should increase when the 510 nm to 540 nm peak decreases, which is not observed in Figure S11.

S13. Fitting of the XB decay kinetics of NPLA and NPLE

The XB kinetics were extracted from the TA data at the XB peak positions. The data of NPLA and NPLE were scaled to the same bleach maximum before global fitting in Igor. The fitting parameters are summarized in Table S3.

Table S3. Fitting parameters of the XB kinetics of NPLA and NPLE

	NPLA	NPLE
c_{e1} /%	18.6 ± 0.4	
τ_{e1} /ps	9.0 ± 0.6	
c_{e2} /%	29.7 ± 1.3	
τ_{e2} /ps	356.4 ± 27.1	1150 ± 75
c_h /%	51.7 ± 1.3	
τ_h /ps	3282 ± 118	6578 ± 194

Assuming that the electron in NPLA decays through the excitonic recombination (with rate constant k_X), and electron trapping ($k_{e,trap}$), the electron (hole) lifetime τ_{e2} can be interpreted as:

$$\frac{1}{\tau_{e2,NPLA}} = k_X + k_{e,trap} \qquad (S5)$$

In NPLE, assuming that certain carrier traps are passivated, the corresponding electron lifetime can be written as:

$$\frac{1}{\tau_{e2,NPLE}} = k_X \qquad (S6)$$

Thus, the lifetime constant of the passivated trapping step can be calculated:

$$\tau_{e,trap} = \frac{1}{k_{e,trap}} = \frac{1}{\frac{1}{\tau_{e2,NPLA}} - \frac{1}{\tau_{e2,NPLE}}} = \frac{\tau_{e2,\,NPLA} \cdot \tau_{e2,\,NPLE}}{\tau_{e2,\,NPLE} - \tau_{e2,\,NPLA}} \qquad (S7)$$

Equation S7 is the expanded version of Equation 2 in the main text.

S14. Estimation of the electron and hole contribution to the XB signal

As shown in Figure S12,[24] the valance band (VB) top in cesium lead bromide perovskite is a mixture of Pb *6s* orbital and Br *4p* orbitals, exhibiting an overall *s* symmetry, resulting in a twofold degenerated VB top. The total angular momentum of the hole (J_h) is 1/2. The conduction band (CB) bottom is contributed by Pb *6p* orbital. With strong spin-orbital-coupling (SOC), the sixfold degeneracy is split into two sets, giving rise to the twofold degenerated CB bottom level with $J_e = 1/2$. The SOC-induced splitting (Δ_{SOC}, ~ 1.56 eV) was estimated to be much larger than the thermal energy at room temperature (26 meV).[25] Given this band edge fine structure, the occupation number of a single electron or hole is calculated to be 1/2, as discussed in our previous report.[19] Thus, the contribution of the electron or hole to the XB signal is estimated to be 50%. Note that the electron-hole exchange interaction splits the fourfold degeneracy of the band edge exciton into one dark state and three bright states.[26] However, the dark state exciton is contributed evenly by the spin-up and spin-down electron or hole [$\frac{1}{\sqrt{2}}(|\Uparrow\rangle|\downarrow\rangle - |\Downarrow\rangle|\uparrow\rangle)$],[24, 26] so the bright-dark splitting has no impact on the electron and hole contribution to XB. For the same reason, the Rashba effect induced by the strong anisotropy of the 2D morphology in NPLs[27] also has no effect on the assignment of the XB contribution.

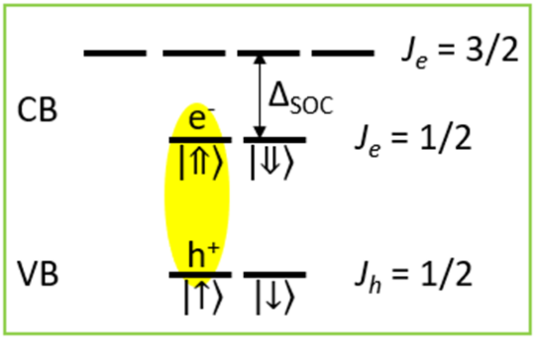

Figure S12. Band edge fine structure of the cesium lead bromide perovskite. A bright exciton (yellow oval) is drawn with the same electron and hole spin.

S15. Kinetics fitting of NPLA-PTZ and NPLE-PTZ

The XB and PTZ^+ PA signal kinetics in NPLA-PTZ are fitted to Equation S8 and S9, respectively.

$$A_{XB,\,NPLA-PTZ}(t) = A'_0\left\{c'_{e1}e^{-\frac{t}{\tau_{e1}}} + c'_h e^{-\frac{t}{\tau'_{HT}}} + c'_{e2}\left[a_1 e^{-\frac{t}{\tau_{e,trap}}} + a_2 e^{-\left(\frac{t}{\tau_{slow}}\right)^{\beta}}\right]\right\}\otimes IRF \qquad (S8)$$

$$A_{PTZ,NPLA}(t) = A_{0,\,PTZ,NPLA}\left[e^{-\frac{t}{\tau'_{HT}}} - e^{-\left(\frac{t}{\tau_{slow}}\right)^{\beta}}\right] \qquad (S9)$$

The first exponential term in Equation S8 represents the fast electron trapping not affected by HT, as discussed in Equation 1. The HT-induced XB decay is represented by the second term with HT time constant τ'_{HT}, which is also the time constant of PTZ^+ growth in Equation S9. The two-phase XB decay after HT is represented by the two terms in the square brackets in Equation S8 with time constant (relative amplitude) of $\tau_{e,trap}$ (a_1) and τ_{slow} (a_2), respectively. The fast decay lifetime $\tau_{e,trap}$ is fixed to be 516 ps, as discussed in the main text. The slow decay lifetime τ_{slow} is applied to account for the long-lived minor XB amplitude, which is further explained by an electron trapping-detrapping model discussed in section S16. Also, this slow decay is linked to the decay of PTZ^+ in Equation S9. A stretched exponential decay was found to appropriately fit the recombination part, likely due to a nonuniform distribution of the electron-hole distance in the CS state, as discussed in the main text. $c'_{e1,2}$ and c'_h are the electron and hole contribution coefficients to XB, A'_0 and $A_{0,\,PTZ,NPL}$ are the maximum amplitudes in each kinetics trace. The global fitting was conducted in Igor Pro 6.3.7.2. The fitting parameters are summarized in Table S4. As shown in Figure 4c, the XB and PTZ^+ kinetics are fitted well by Equation S8 and S9, respectively. Remarkably, the fitted HT-induced XB decay amplitude c'_h (47.0 ± 6.7%) is close to the c_h (51.7 ± 1.3%) in free NPLs, suggesting that most of the excited holes are transferred to PTZ, consistent with the near unity PL quenching by HT.

Table S4. Fitting parameters of the XB and scaled PTZ^+ kinetics in NPLA-PTZ.

	XB	PTZ^+
A'_0/mOD	17.7 ± 0.3	8.0 ± 0.4
c'_{e1}	15.4 ± 3.5 %	
τ_{e1}/ps	9.0	
c'_h	47.6 ± 3.7 %	
τ'_{HT}/ps	60.0 ± 5.4	60.0 ± 5.4
c'_{e2}	37.0 ± 3.7 %	
$T_{e,trap}$/ps (a_1)	516 (80.6 ± 3.4 %)	
τ_{slow}/ns (a_2)	42.8 ± 14.6 (19.4 ± 3.4 %)	42.8 ± 14.6
β	0.19 ± 0.01	0.19 ± 0.01

The XB and PTZ^+ kinetics in NPLE-PTZ in Figure 4d are fitted to Equation S10 and S11, respectively. The XB decay is fitted to the sum of the initial fast electron trapping as in pure NPLs (relative amplitude c''_{e1}, lifetime constant τ_{e1}), the hole transfer (c''_h, τ''_{HT}), and the electron recombination with PTZ^+

(c''_{e2}, τ''_{CR}). Biexponential decay was found necessary to fit the electron recombination part. For the PTZ^+ kinetics (Equation S11), the growth and decay parts are linked to the hole transfer and the electron recombination in Equation S10, respectively. Different from the fitting of NPLA-PTZ (Equation S8), the electron trapping lifetime $\tau_{e,trap}$ is not included in the decay of XB in NPLE-PTZ (Equation S10). Fitting parameters are summarized in Table S5.

$$A_{XB,\,NPLE-PTZ}(t) = A''_0\left\{c''_{e1}e^{-\frac{t}{\tau_{e1}}} + c''_h e^{-\frac{t}{\tau''_{HT}}} + c''_{e2}\left[b_1 e^{-\frac{t}{\tau''_{CR1}}} + b_2 e^{-\frac{t}{\tau''_{CR2}}}\right]\right\}\otimes IRF \qquad (S10)$$

$$A_{PTZ,NPLE}(t) = A_{0,\,PTZ,NPLE}\left[e^{-\frac{t}{\tau''_{HT}}} - \left(b_1 e^{-\frac{t}{\tau''_{CR1}}} + b_2 e^{-\frac{t}{\tau''_{CR3}}}\right)\right] \qquad (S11)$$

Table S5. Fitting parameters of the XB and scaled PTZ^+ kinetics in NPLE-PTZ.

	XB	PTZ^+
A''_0/mOD	11.7 ± 0.2	5.6 ± 0.4
c''_{e1}	10.0 ± 4.1 %	
τ_{e1}/ps	9.0	
c''_h	48.3 ± 4.1 %	
τ''_{HT}/ps	78.4 ± 8.1	78.4 ± 8.1
c''_{e2}	41.7 ± 4.1 %	
τ''_{CR1}/ns (b_1)	2.0 ± 0.2 (83.7 ± 1.6 %)	2.0 ± 0.2 (83.7 ± 1.6 %)
τ''_{CR2}/ns (b_2)	24.1 ± 11.8 (16.3 ± 1.6 %)	
τ''_{CR3}/µs (b_2)		19.7 ± 11.7 (16.3 ± 1.6 %)

Note that the lifetime constant of the second exponential decay ($b_2 = 16.3\ \pm 1.6\%$) of PTZ^+ (τ''_{CR3}) is different from that in Equation S10 (τ''_{CR2}). As will be seen in section S16, the difference of this minor portion of the charge recombination may be caused by the trapping-detrapping behavior of the CB electron in the minor part of NPLEs.

S16. The electron trapping-detrappping model

A careful examination of the long-lived minor XB signal in NPLA-PTZ reveals that this part decays together with the PTZ^+ PA signal, as shown in Figure S13a and Figure S10a inset, where the PTZ^+ kinetics is rescaled to match the XB amplitude at longer delay times (> 1 ns). This observation can be explained by an electron trapping-detrapping model, as shown in Figure S13b. In this model, the CB electron decays through trapping and recombination with PTZ^+ and can be replenished through detrapping from the trap state. It is also assumed that the direct charge recombination of trapped electrons with PTZ^+

is much slower and can be neglected. The rate constants of CR, trapping and detrapping are denoted as k_1, k_2, and k_3, respectively, and k_2 is assumed to be much larger than k_1 and k_3.

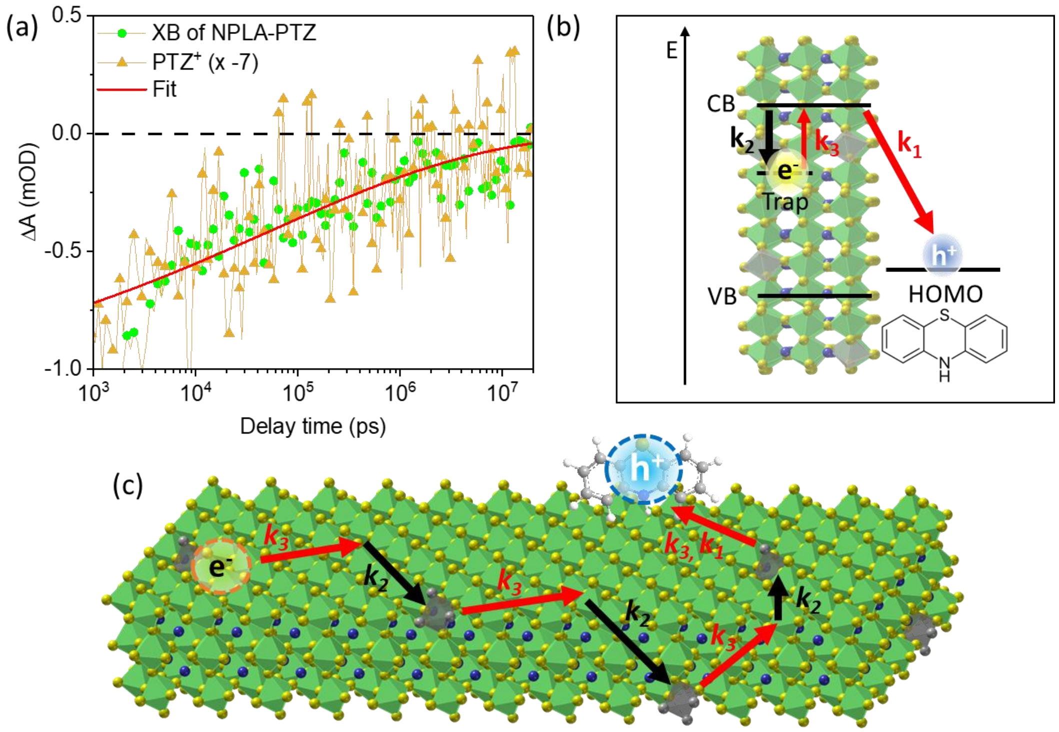

Figure S13. (a) A zoom-in view of the XB kinetics of NPLA-PTZ in Figure 4c from 1 ns to 20 μs. PTZ^+ kinetics is flipped and scaled by 7 times to compare with the XB kinetics. The red line is the fit to the PTZ^+ kinetics. (b) Energetic diagram of the electron trapping-detrapping model in the CS state. The electron can transfer between the CB and trap state and can only recombine with hole in PTZ^+ from CB. (c) Illustration of electron hopping between Pb^{2+} vacancies through trapping (k_2)-detrapping (k_3) before final recombination with PTZ^+ (k_1).

Let N_{CB} and N_T represent the number of electrons on CB and in trap state as a function of delay time t. The differential rate equation for N_{CB} and N_T can be written as:

$$\frac{dN_{CB}}{dt} = -k_2 N_{CB} - k_1 N_{CB} + k_3 N_T \qquad (S12)$$

$$\frac{dN_T}{dt} = k_2 N_{CB} - k_3 N_T \qquad (S13)$$

To solve the rate equation of N_{CB}, the second order differential equation of N_{CB} is written and terms related to N_T are replaced:

$$\frac{d^2N_{CB}}{dt^2} = -(k_1+k_2)\frac{dN_{CB}}{dt} + k_3\frac{dN_T}{dt} = -(k_1+k_2)\frac{dN_{CB}}{dt} + k_3(k_2N_{CB} - k_3N_T)$$
$$= -(k_1+k_2+k_3)\frac{dN_{CB}}{dt} - k_1k_3N_{CB} \qquad (S14)$$

Rearranging Equation S14 gives:

$$\frac{d^2N_{CB}}{dt^2} + (k_1+k_2+k_3)\frac{dN_{CB}}{dt} + k_1k_3N_{CB} = 0 \qquad (S15)$$

The general solution to Equation S15 is:[28]

$$N_{CB} = a_1e^{-k_{fast}t} + a_2e^{-k_{slow}t} \qquad (S16)$$

Equation S16 indicates that the decay kinetics of CB electrons can be described by a two exponential decay function. The pre-exponential factors a_1 and a_2 are not analytically solved. As shown in the fitting results in Table S4 and discussed below, the *normalized* a_1 and a_2 are fitted to be 80.6 ± 3.4 % and 19.4 ± 3.4 %, respectively. The rate constant k_{fast} and k_{slow} are given by:

$$k_{fast} = \frac{1}{2}(k_1+k_2+k_3)\left[1+\sqrt{1-\frac{4k_1k_3}{(k_1+k_2+k_3)^2}}\right] \qquad (S17)$$

$$k_{slow} = \frac{1}{2}(k_1+k_2+k_3)\left[1-\sqrt{1-\frac{4k_1k_3}{(k_1+k_2+k_3)^2}}\right] \qquad (S18)$$

From the assumption of $k_2 \gg k_3$ and $k_2 \gg k_1$, it is known that

$$\frac{4k_1k_3}{(k_1+k_2+k_3)^2} \ll 1 \qquad (S19)$$

Then the Taylor expansion can be applied to the square root term in Equation S17 and S18:

$$\sqrt{1-\frac{4k_1k_3}{(k_1+k_2+k_3)^2}} \approx 1 - \frac{1}{2}\frac{4k_1k_3}{(k_1+k_2+k_3)^2} \qquad (S20)$$

The decay rate constants can thus be written as:

$$k_{fast} = (k_1+k_2+k_3) - \frac{k_1k_3}{k_1+k_2+k_3} \qquad (S21)$$

$$k_{slow} = \frac{k_1k_3}{k_1+k_2+k_3} \approx \frac{k_3}{k_2}k_1 \qquad (S22)$$

Equation S21 shows that the fast decay rate constant k_{fast} is mainly contributed by the electron trapping rate constant k_2. In Equation S22, since $k_2 \gg k_3$, k_{slow} is much smaller than k_1. This kinetics model yields a two-phase decay of the electron population on the CB: one fast decay and one slow decay, which can account for the XB decay kinetics in NPLA-PTZ after HT. In the fitting of the NPLA-PTZ XB

kinetics (Equation S8), $\frac{1}{\tau_{e,trap}}$ and $\frac{1}{\tau_{slow}}$ can be regarded as k_{fast} and k_{slow}, respectively, and the relative amplitude a_1 and a_2 (in Equation S8 Table S4) can be used in Equation S16.

The decay of PTZ^+ in NPLA-PTZ can be written as:

$$\frac{dN_{PTZ^+}}{dt} = -k_1 N_{CB} = -k_1\left(a_1 e^{-k_{fast}t} + a_2 e^{-k_{slow}t}\right) \qquad (S23)$$

Integration of Equation S23 gives:

$$N_{PTZ^+} = \frac{k_1 a_1}{k_{fast}} e^{-k_{fast}t} + \frac{k_1 a_2}{k_{slow}} e^{-k_{slow}t} \qquad (S24)$$

Under the assumption that $k_2 \gg k_3$ and $k_2 \gg k_1$, a comparison of the two pre-exponential factors indicates that the decay of N_{PTZ^+} is dominated by the second exponential decay term:

$$\frac{(\frac{k_1 a_1}{k_{fast}})}{(\frac{k_1 a_2}{k_{slow}})} = \frac{a_1}{a_2}\frac{k_{slow}}{k_{fast}} = \frac{80.6\%}{19.4\%}\frac{(\frac{k_1 k_3}{k_1 + k_2 + k_3})}{(k_1 + k_2 + k_3) - \frac{k_1 k_3}{k_1 + k_2 + k_3}} = \frac{4}{\frac{(k_1 + k_2 + k_3)^2}{k_1 k_3} - 1} \ll 1 \qquad (S25)$$

Thus:

$$N_{PTZ^+} \approx \frac{k_1 a_2}{k_{slow}} e^{-k_{slow}t} \qquad (S26)$$

Equation S26 thus explains the same decay kinetics between PTZ^+ and the long-lived minor XB in NPLA-PTZ shown in Figure S13a and Figure S10a inset. In addition, Equation S22 and S26 also explain the much slower decay of the PTZ^+ in NPLA-PTZ when compared to the PTZ^+ in NPLE-PTZ. In NPLA-PTZ, the PTZ^+ decays with the rate constant k_{slow}, while most of the PTZ^+ in NPLE-PTZ is believed to decay with the rate constant k_1.

Note that the long tail of the PTZ^+ kinetics in NPLE-PTZ in Figure 4d is attributed to similar trapping-detrapping of electrons in NPLE but with largely reduced percentage (16 % of the remaining electrons after HT, see Table S5 for fitting parameters), and the corresponding long tail of XB is not observed probably due to the small amplitude.

The initial fast trapped electron before HT ($\tau_{e1} = 9.0\ \pm 0.6\ ps$, Equation 1) is not involved in the trapping-detrapping model. Since the PTZ^+ kinetics shows no extra detectable decay components in NPLA-PTZ, the recombination of this type of trapped electrons with PTZ^+ is included in the stretched exponential decay in Equation S8 and S9.

This electron trapping-detrapping model also implies that the trapped electron may approach to the PTZ^+ by hopping between the trap states (or Pb^{2+} vacancies), as illustrated in Figure S13c. After detrapping (k_3), the CB electron can be trapped (k_2) near a different Pb^{2+} vacancy before it recombines with PTZ^+ (k_1). This process may happen multiple times until the electron is close enough to the PTZ^+ cation to favor the electron-hole recombination. An NPL-lateral-size-dependent HT experiment or a trap-density-dependent HT experiment may give more insights into this electron hopping picture.

S17. Estimation of the detrapping lifetime and trap depth

Equation S22 is used to estimate the detrapping lifetime (${k_3}^{-1}$) in Figure S13b. The trapping lifetime (${k_2}^{-1}$) is taken as 500 ps, as discussed in section S16. The CB-to-HOMO transfer lifetime (${k_1}^{-1}$) is taken as the half-lifetime of the CR in NPLE-PTZ, 2.1 ns. The lifetime corresponding to ${k_{slow}}^{-1}$ is taken as the CR half-lifetime in NPLA-PTZ, 68 ns. As a result, the detrapping lifetime ${k_3}^{-1}$ is calculated to be 16 ns.

The trap depth (ΔE) is estimated by assuming a Boltzmann distribution of the electrons among the two levels: CB and trap. It is further assumed that the relative amplitude of the slow decay part of XB in NPLA-PTZ (a_2 in Equation S8) represents the amount of electrons on CB, and the relative amplitude of the fast decay part of XB (a_1 in Equation S8) represents the amount of electrons in trap. Under Boltzmann distribution:

$$\frac{a_1}{a_2} = e^{\frac{\Delta E}{kT}} \qquad (S27)$$

In Equation S27, k is the Boltzmann constant and T is the room temperature. The fitted a_1 and a_2 values in Table S4 gives a ΔE of 36 meV. If the relative amplitude (${c'}_{e1}$) of the initial fast trapped electron ($\tau_{e1} = 9\ ps$ in Equation 8) is also counted as the population in the trap state, then the equilibrium between the trap state and the CB can be written as:

$$\frac{{c'}_{e1} + {c'}_{e2} \cdot a_1}{{c'}_{e2} \cdot a_2} = e^{\frac{\Delta E}{kT}} \qquad (S28)$$

Equation S28 gives a ΔE of 46 meV with the fitted parameters in Table S4. A detailed study of this trap state may be facilitated by a temperature dependent HT experiment[29] and related structural characterization or computation.

Reference

1. Gramlich, M.; Lampe, C.; Drewniok, J.; Urban, A. S., How Exciton-Phonon Coupling Impacts Photoluminescence in Halide Perovskite Nanoplatelets. *J Phys Chem Lett* **2021**, 11371-11377.
2. Bohn, B. J.; Tong, Y.; Gramlich, M.; Lai, M. L.; Doblinger, M.; Wang, K.; Hoye, R. L. Z.; Muller-Buschbaum, P.; Stranks, S. D.; Urban, A. S.; Polavarapu, L.; Feldmann, J., Boosting Tunable Blue Luminescence of Halide Perovskite Nanoplatelets through Postsynthetic Surface Trap Repair. *Nano Lett* **2018,** *18* (8), 5231-5238.
3. Wu, Y.; Wei, C.; Li, X.; Li, Y.; Qiu, S.; Shen, W.; Cai, B.; Sun, Z.; Yang, D.; Deng, Z.; Zeng, H., In Situ Passivation of PbBr64– Octahedra toward Blue Luminescent CsPbBr3 Nanoplatelets with Near 100% Absolute Quantum Yield. *ACS Energy Letters* **2018,** *3* (9), 2030-2037.
4. Do, M.; Kim, I.; Kolaczkowski, M. A.; Kang, J.; Kamat, G. A.; Yuan, Z.; Barchi, N. S.; Wang, L. W.; Liu, Y.; Jurow, M. J.; Sutter-Fella, C. M., Low-dimensional perovskite nanoplatelet synthesis using in situ photophysical monitoring to establish controlled growth. *Nanoscale* **2019,** *11* (37), 17262-17269.
5. Ghribi, A.; Ben Aich, R.; Boujdaria, K.; Barisien, T.; Legrand, L.; Chamarro, M.; Testelin, C., Dielectric Confinement and Exciton Fine Structure in Lead Halide Perovskite Nanoplatelets. *Nanomaterials (Basel)* **2021,** *11* (11).
6. Reynolds, G. A.; Drexhage, K. H., New coumarin dyes with rigidized structure for flashlamp-pumped dye lasers. *Optics Communications* **1975,** *13* (3), 222-225.
7. Zhu, H.; Song, N.; Rodriguez-Cordoba, W.; Lian, T., Wave Function Engineering for Efficient Extraction of up to Nineteen Electrons from One CdSe/CdS Quasi-Type II Quantum Dot. *Journal of the American Chemical Society* **2012,** *134* (9), 4250-4257.
8. Li, Q.; Lian, T., Ultrafast Charge Separation in Two-Dimensional CsPbBr3 Perovskite Nanoplatelets. *J Phys Chem Lett* **2019**.
9. Lin, K.; Xing, J.; Quan, L. N.; de Arquer, F. P. G.; Gong, X.; Lu, J.; Xie, L.; Zhao, W.; Zhang, D.; Yan, C.; Li, W.; Liu, X.; Lu, Y.; Kirman, J.; Sargent, E. H.; Xiong, Q.; Wei, Z., Perovskite light-emitting diodes with external quantum efficiency exceeding 20 per cent. *Nature* **2018,** *562* (7726), 245-248.
10. Liang, J.; Wang, C.; Wang, Y.; Xu, Z.; Lu, Z.; Ma, Y.; Zhu, H.; Hu, Y.; Xiao, C.; Yi, X.; Zhu, G.; Lv, H.; Ma, L.; Chen, T.; Tie, Z.; Jin, Z.; Liu, J., All-Inorganic Perovskite Solar Cells. *J Am Chem Soc* **2016,** *138* (49), 15829-15832.
11. Huang, J.; Huang, Z.; Jin, S.; Lian, T., Exciton Dissociation in CdSe Quantum Dots by Hole Transfer to Phenothiazine. *J Phys Chem C* **2008,** *112* (49), 19734-19738.
12. Matylitsky, V. V.; Dworak, L.; Breus, V. V.; Basche, T.; Wachtveitl, J., Ultrafast charge separation in multiexcited CdSe quantum dots mediated by adsorbed electron acceptors. *J Am Chem Soc* **2009,** *131* (7), 2424-5.
13. Mandal, S.; George, L.; Tkachenko, N. V., Charge transfer dynamics in CsPbBr3 perovskite quantum dots-anthraquinone/fullerene (C60) hybrids. *Nanoscale* **2019,** *11* (3), 862-869.
14. Montalti, M.; Credi, A.; Prodi, L.; Gandolfi, M. T., *Handbook of photochemistry*. CRC press: 2006.
15. Luo, X.; Liang, G.; Han, Y.; Li, Y.; Ding, T.; He, S.; Liu, X.; Wu, K., Triplet Energy Transfer from Perovskite Nanocrystals Mediated by Electron Transfer. *J Am Chem Soc* **2020,** *142* (25), 11270-11278.
16. Luo, X.; Han, Y.; Chen, Z.; Li, Y.; Liang, G.; Liu, X.; Ding, T.; Nie, C.; Wang, M.; Castellano, F. N.; Wu, K., Mechanisms of triplet energy transfer across the inorganic nanocrystal/organic molecule interface. *Nat Commun* **2020,** *11* (1), 28.
17. Weidman, M. C.; Goodman, A. J.; Tisdale, W. A., Colloidal Halide Perovskite Nanoplatelets: An Exciting New Class of Semiconductor Nanomaterials. *Chemistry of Materials* **2017,** *29* (12), 5019-5030.
18. Kobosko, S. M.; DuBose, J. T.; Kamat, P. V., Perovskite Photocatalysis. Methyl Viologen Induces Unusually Long-Lived Charge Carrier Separation in CsPbBr3 Nanocrystals. *ACS Energy Letters* **2019,** *5* (1), 221-223.

19. He, S.; Li, Q.; Jin, T.; Lian, T., Contributions of exciton fine structure and hole trapping on the hole state filling effect in the transient absorption spectra of CdSe quantum dots. *The Journal of Chemical Physics* **2022,** *156* (5), 054704.
20. Castaneda, J. A.; Nagamine, G.; Yassitepe, E.; Bonato, L. G.; Voznyy, O.; Hoogland, S.; Nogueira, A. F.; Sargent, E. H.; Cruz, C. H.; Padilha, L. A., Efficient Biexciton Interaction in Perovskite Quantum Dots Under Weak and Strong Confinement. *ACS Nano* **2016,** *10* (9), 8603-9.
21. Bertolotti, F.; Nedelcu, G.; Vivani, A.; Cervellino, A.; Masciocchi, N.; Guagliardi, A.; Kovalenko, M. V., Crystal Structure, Morphology, and Surface Termination of Cyan-Emissive, Six-Monolayers-Thick CsPbBr3 Nanoplatelets from X-ray Total Scattering. *ACS Nano* **2019,** *13* (12), 14294-14307.
22. Maes, J.; Balcaen, L.; Drijvers, E.; Zhao, Q.; De Roo, J.; Vantomme, A.; Vanhaecke, F.; Geiregat, P.; Hens, Z., Light Absorption Coefficient of CsPbBr3 Perovskite Nanocrystals. *J Phys Chem Lett* **2018,** *9* (11), 3093-3097.
23. Alkaitis, S. A.; Beck, G.; Graetzel, M., Laser photoionization of phenothiazine in alcoholic and aqueous micellar solution. Electron transfer from triplet states to metal ion acceptors. *Journal of the American Chemical Society* **1975,** *97* (20), 5723-5729.
24. Becker, M. A.; Vaxenburg, R.; Nedelcu, G.; Sercel, P. C.; Shabaev, A.; Mehl, M. J.; Michopoulos, J. G.; Lambrakos, S. G.; Bernstein, N.; Lyons, J. L.; Stoferle, T.; Mahrt, R. F.; Kovalenko, M. V.; Norris, D. J.; Raino, G.; Efros, A. L., Bright triplet excitons in caesium lead halide perovskites. *Nature* **2018,** *553* (7687), 189-193.
25. Zhang, X.; Shen, J. X.; Van de Walle, C. G., First‐Principles Simulation of Carrier Recombination Mechanisms in Halide Perovskites. *Advanced Energy Materials* **2019,** *10* (13).
26. Sercel, P. C.; Lyons, J. L.; Wickramaratne, D.; Vaxenburg, R.; Bernstein, N.; Efros, A. L., Exciton Fine Structure in Perovskite Nanocrystals. *Nano Lett* **2019,** *19* (6), 4068-4077.
27. Gramlich, M.; Swift, M. W.; Lampe, C.; Lyons, J. L.; Doblinger, M.; Efros, A. L.; Sercel, P. C.; Urban, A. S., Dark and Bright Excitons in Halide Perovskite Nanoplatelets. *Adv Sci (Weinh)* **2022,** *9* (5), e2103013.
28. Rukmangadachari, E., *Mathematical methods*. Pearson Education India: 2009.
29. Olshansky, J. H.; Balan, A. D.; Ding, T. X.; Fu, X.; Lee, Y. V.; Alivisatos, A. P., Temperature-Dependent Hole Transfer from Photoexcited Quantum Dots to Molecular Species: Evidence for Trap-Mediated Transfer. *ACS Nano* **2017,** *11* (8), 8346-8355.
